# Exploring Meal and Snacking Behaviour of Older Adults in Australia and China

**DOI:** 10.3390/foods9040426

**Published:** 2020-04-03

**Authors:** Behannis Mena, Hollis Ashman, Frank R. Dunshea, Scott Hutchings, Minh Ha, Robyn D. Warner

**Affiliations:** School of Agriculture and Food, Faculty of Veterinary and Agricultural Sciences, University of Melbourne, Parkville, VIC 3010, Australia; hollis.ashman@unimelb.edu.au (H.A.); fdunshea@unimelb.edu.au (F.R.D.); scott.c.hutchings@outlook.com (S.H.); minh.ha@unimelb.edu.au (M.H.); robyn.warner@unimelb.edu.au (R.D.W.)

**Keywords:** focus group, meat products, qualitative multivariate analysis, conjoint analysis, older adults

## Abstract

Sensory perception and food preferences change as we age. This paper encompassed two studies with the aim being to investigate meal and snacking behaviour of older adults towards food, especially meat products, and understand the desirable characteristics of those products. A qualitative multivariate analysis (QMA) focus group with Australian and Chinese older (60–81 years old) adults was conducted. A conjoint concept database was used to determine older consumers’ wants and needs for food in Australia and China. The QMA suggested that Australian consumers are not eating a proper breakfast or dinner but are ‘snacking’ throughout the day. In contrast, Chinese consumers are eating three regular meals through the day and occasionally snacks. For both groups, texture and flavour were key drivers for food choice. Difficulty in eating meat products was evident, e.g., beef jerky was found too dry and hard. Older consumers in China and Australia differed in responses to the four food categories investigated in terms of product traits and segmentation. Both the conjoint analysis and QMA showed that demographics have an impact on consumer preferences towards food. This research suggested that there is an opportunity to create ready-to-eat, nutrient dense products to enhance the wellness of older consumers.

## 1. Introduction

In 2017, 8.7% of the population worldwide (654 million) were aged ≥65 [1]. This is expected to grow to nearly 17% (1.6 billion) of the worldwide population by 2050 [2]. With reduced fertility rates and people living longer, this is becoming a major concern for many European, Asian and North American countries [2]. For Australia, the group of people aged ≥65 was 15.7% in 2018 [3]. Life expectancy is increasing as the world population is getting older [4], thus lifestyle factors become increasingly relevant to improve the quality of the later years in life.

While Australia has a higher healthy life expectancy than China, it has been well established that general heath can decline as we age; and there are many elements that influence this fact. One of them is nutrition, as it is important to maintain healthy eating habits throughout life. Declining energy intake in older adults is an important phenomenon to address. Dietary patterns in older adults have received little attention [5]. Too often, older adults’ nutrition practices are ignored, which impede health maximisation and quality of life improvement [5]. Good nutrition is essential to slow the mental and physical decline of ageing people. Sarcopenia, the degenerative loss of skeletal muscle mass and strength associated with ageing [6], is an important condition to consider. Research has demonstrated that increased protein in the diet of older people, coupled with exercise, can significantly delay sarcopenia [7]. One approach to increasing protein intake in older people is through the consumption of high protein meat-based snacks. To develop red meat-based snacks, the starting point is to understand ‘snacking’. In particular, why people snack, the underlying motivations that lead them to snack and shape the choices they make and the most relevant channels through which a snack would be sourced to satisfy the need. Using these foundations will enable the design of superior red meat snack products that better meet these criteria [8].

Qualitative research is often used to develop and refine hypotheses in product development, which allows for quick, inexpensive probing of consumer demands in a natural and comfortable environment [9,10]. Qualitative multivariate analysis (QMA) and conjoint analysis are two powerful methods to investigate consumers´ choices. Studies applying those techniques to understand Australian and Chinese older people preferences towards meat products have not been conducted. QMA fosters a more ‘bottom-up’ approach to product development as new products can be more specifically tailored to the attributes seen as positive and negative by particular market segments. The advantage of conjoint analysis over other more direct approaches of analysis (i.e., asking consumers what they like and dislike) is that it provides an accurate analysis of the consumer mind rather than the factors that consumers ‘think’ affect their decisions.

The aim of this research was therefore to use QMA and conjoint analysis to investigate food preferences and behaviours of older Australian and Chinese adults and understand how they differ. Some of the questions we hoped to answer were; (i) how do snacks fit with elder consumers’ preferences? (ii) is what we know regarding older adults eating behaviour correct? (iii) what else do we need to know? Another aim was to understand the meat market from older people’s perspective, to understand what is similar/dissimilar among older adults and to find gaps where possible products could be developed that do not currently exist in the market.

## 2. Materials and Methods

### 2.1. QMA Study

QMA is a relatively new technique that has been used mainly in industry settings [11]. This is a reliable technique to perform research exploration. Drake et al. [11] found that the results obtained from quantitative mapping techniques (*n* = 110 consumers), i.e., landscape segmentation analysis with agglomerative hierarchical clustering, were similar to those obtained through QMA with a smaller sample size (*n* = 12 consumers). The QMA technique used by [11] (also known as perceptual mapping, landscape mapping, Napping^®^) uses a similar protocol to this study with group discussions and mapping exercise which brings advantages from integration of many techniques. Through QMA, researchers can discover insights about the products directly from consumers without any predetermined agenda, discussing only important matters identified in the discussion [11,12].

Exploratory studies were carried out in Australia at the Hastings Lifestyle Retirement Village with Australian participants in Hastings, Victoria and at the Chinese Senior Citizens Club with Chinese participants in Frankston, Victoria. QMA with focus group methodology was used, following a semi-structured protocol and was conducted under the University of Melbourne human ethics protocol number 1749295.

#### 2.1.1. Participants

Sixteen (13 female, 3 male) Australian meat consumers, who were selected for having active lifestyles and being 65–79 years old, were recruited for two focus groups (*n* = 8 in each focus group). Twenty-one (17 female, 4 male) Chinese meat consumers with active lifestyles and being 60–81 years old, participated in two focus groups (*n* = 12 and 9). Due to limited number of participants, it was difficult to conduct this trial with balance for gender. The ratio of 4:1, female:male can be justified by the fact that women generally live longer than males [13] and they were more eager to participate in the focus groups. Participants declared they were not taking any strong medication and did not have any health conditions that would influence taste, smell or chewing of samples except for one participant that declared cream, onion and garlic allergies and abstained from tasting the bolognese. The sample size was determined according to saturation principles [14]. Participants were recruited by staff of the retirement village and senior club. All participants gave informed consent, signed allergen forms and received a small compensatory gift. A plain language statement was given with detailed information of the study.

#### 2.1.2. Procedure

Data were collected in three stages following the sequence below and each focus group went through the three stages sequentially, over a period of 2 h. Both focus groups within a demographic, were conducted on the same day, one after the other. Sessions for each demographic group were conducted on separate dates. For the Chinese focus groups, a translator facilitated the activity and all paper materials were translated into Chinese. Sessions had video and voice recordings made. A discussion guide (Table S1) was followed to facilitate conversation and reach the learning objectives.

Stage 1: A 5Ws table (When, What, Why, Where, Who and What With) documenting the participant’s meal/snacking behaviour was completed. Participants were asked to describe their eating habits throughout the day (early morning, breakfast, mid-morning, lunch, afternoon, dinner and after dinner) using a customised list of meat products sensory descriptors for flavour, appearance, texture and aroma (Table S2). These descriptors were given to enhance the flow of conversation while their responses were recorded on a flip chart.

**Figure 1:**
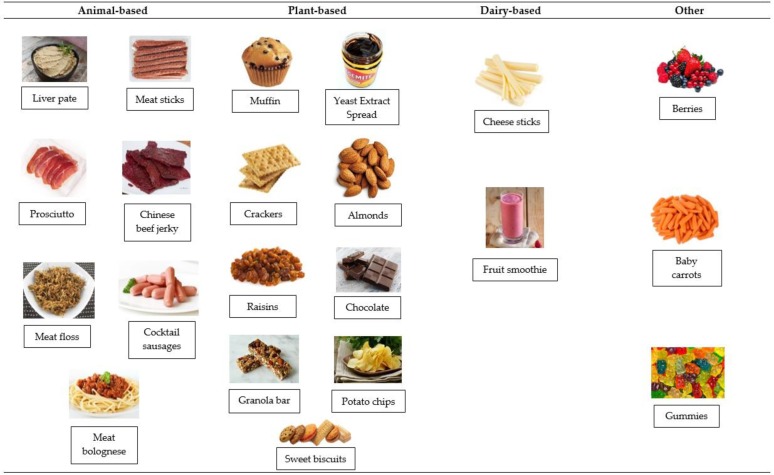
Images of the snacks presented in the perpetual mapping stage, and grouped into animal-, plant-, dairy-based and other.

Stage 2: Tasting of seven commercially available meat products (pictorial reference is shown in Figure 1) including meat bolognese (pasta and minced meat), cocktail sausage, prosciutto, meat sticks, meat floss (light and fluffy shredded dry pork common in China), liver pate and Chinese beef jerky (softer and more moist than traditional beef jerky produced and consumed in Australia). These products were chosen to provide stimuli across a range of textures (softest texture was liver pate and hardest was beef jerky) and familiarity (least familiar for Australians was meat floss, for Chinese was liver pate and most familiar for Australians was cocktail sausages, for Chinese was meat floss). Participants were presented with products, in a randomised sequence according to demographic group, everyone ate the same product at the same time. For each product, participants completed a paper-based questionnaire with open response questions regarding attributes of appearance, texture contrast, flavour/aroma. Products were purchased at local stores and kept under refrigeration (5 °C) where required. Cocktail sausages were cooked with boiling water and meat bolognese was heated in a microwave before serving. The remaining samples were ready-to-eat. Serving size was about 30 g per sample and they were served individually.

Stage 3: Perceptual mapping of commonly consumed snack foods was conducted, which consisted of providing pictures and placing them on a two-dimensional map with the x-axis being: ‘Everyday’ to ‘Indulgent’ and *Y*-axis being ‘Likely to Eat’ to ‘Less Likely to Eat’. Products were selected from a wide range of texture, protein source and popularity (Figure 1). The map was drawn on a flipchart paper glued to a table with x- and y-axes taped on it using sticky notes. Participants sat around a large table in a private room, a moderator trained in QMA techniques stood in front of them to facilitate discussion and three assistants sat outside the group, taking notes. At the start of the session, a picture of cocktail sausages was shown and placed in the middle of the map as the reference point. This product was used as starting point for being generic (safe/popular), thus forcing participants to use the entire space and avoid clumping pictures in one quadrant and helping to separate out the differences between products more easily. The analysis involved foods being grouped by identifying common characteristics among them within a quadrant.

### 2.2. Conjoint Study

Conjoint measurement is “a simple descriptive method, namely the use of experimental design to understand reactions to ideas by measuring reactions to mixtures of ideas” [15]. Conjoint measurement uses experimental design, mixing, together small components (‘idealets’), generating combinations, acquiring subjective responses to those combinations and then deducing what components drive the reactions [16]. The point of view of conjoint measurement in particular, and statistical experimental design in general, is that the combination of independent variables allows each of them to affect the other in a way that could not be seen in the traditional one-at-a-time approach [17]. This technique allows us to better understand the mind of the consumer to generate insights and product ideas and, also provides various outputs for analysis, including part-worth utilities, counts, importance by ranking, shares of preference and purchase likelihood simulations [18]. Furthermore, conjoint analysis involves providing consumers with a set of product descriptions and requiring them to rate whether each description would or would not appeal to them [19]. These product descriptions comprise a core set of elements, which are systematically varied among the descriptions by a “mix and match” method. Once the behaviour of individuals is understood, consumers can be grouped according to their patterns of utility value also known as regression coefficients.

#### 2.2.1. Selection of Foods and Respondents

The concept database comprised 18 food and beverage categories (wine, coffee, chocolate, yogurt, milk drinks, ice cream, cheese, baby formula, capsicum, pumpkin, tomato, banana, melon, tea, grains, beef, pork, olive oil) for consumers and was conducted in both Australia and China. The original objective of the database was to understand the key levers that drive ‘premiumness’ across those food groups as representations of the overall food industry. Both Australian and Chinese consumers were included in order to understand both the domestic market and potential export markets. In addition, an understanding of cultural bias is provided by comparing the two cohorts. For the current research, the focus was on beef, pork, chocolate and cheese categories, as according to the QMA study described above, these categories represent the key categories older consumers self-report eating. A total of 14,400 consumers (7200 in Australia; 7200 in China) were surveyed online and about 400 consumers per category were achieved. Recruitment was based on general population criteria, i.e., age, gender and geographical location that were representative of the overall demographic of the country. The survey had a male/female split of 43/57 for Australia and 51/49 for China to allow for gender segmentation of the data and was translated into Mandarin by an experienced translator for distribution in China and to ensure clear cross-country comparisons. Translation and data collection were conducted by Research Now Pty Ltd. (currently known as DynataTM). Respondents resided in both urban and rural areas and had familiarity with the food category evaluated. Each respondent only answered questions within one category.

#### 2.2.2. Key Attributes

There were six key attributes evaluated for each category: product, packaging, ingredient uniqueness, provenance process, channel and occasion. The design of the experiment allowed for each of these attributes to have six levels ranging from a very basic everyday description to a very premium description (Table S3). Respondents were presented with different concepts, generated randomly from the possible combination of factors (6 attributes, 6 levels) and representing different statistically derived variations of the product concepts. They were required to choose which was considered most, or least, premium. The concepts were couched in language that goes beyond the simple one- or two-word statements into much more descriptive terms [20] and Table S3 shows a description of the terms used. To improve the consumers’ response and achieve a better grasp of the consumers mind-set in response to the product, instead of using a simple one- or two-word statement, the concepts were more detailed to avoid confusion [20]. In addition, key questions about being the first to buy or adopt new products or ideas (Table S4), were asked for stratification of the consumers into segments based on adoption characteristics (see data analysis section for detail). 

#### 2.2.3. Conjoint Design

Conjoint analysis is a survey-based statistical technique used in market research that helps determine how people value different attributes that make up an individual product or service. The objective of conjoint analysis is to determine what combination of a limited number of attributes is most influential in respondent choice or decision making. In our study, six attributes along with pictures of packaging concepts for different products were shown to survey respondents (Figure S1). We then analysed how the respondents made preferences between these products, thus the implicit value (utility or part-worth) of the individual elements making up the product or service was determined. In this study, each of six attributes can be broken down into six levels. Both attributes and levels were defined based on previous marketing research and researcher’s knowledge. For instance, the levels for ingredient uniqueness include basic, safe ingredients, high quality ingredients, chef inspired ingredients, bush ingredients, limited seasonal ingredients and healthy ingredients. Each of was specific to the food category under consideration and details are shown in Table S3.

#### 2.2.4. Experimental Design

Similar to the method of Foley [20], we invited the respondents to participate, by sending an email. Those who agreed to participate simply clicked on a link embedded in the e-mail invitation. They were then taken to a survey ‘wall’, which listed the available food categories. Respondents ticked one box per food category, and the software kept track of how often different levels for each attribute were chosen. Then, with regression analysis, coefficients were obtained which is where the data presented in this paper came from.

Respondents were shown a set of 4 concepts to choose from (Figure S1) for each category. In total, 40 concepts per food category were used, created from a combination of levels from all of the constituent attributes. Respondents were asked to rate the products in terms of premiumness in a partial factorial design. Each concept was composed of a unique combination of levels. The data consisted of individual ratings among alternative combinations that were shuffled randomly for consumers. As the number of combinations of attributes and levels within food category increase, the number of potential concepts also increase exponentially. Consequently, fractional factorial from 6 × 6 design was used to reduce the number of concepts that had to be evaluated, while ensuring enough data are available for statistical analysis, resulting in a carefully controlled set of ‘concepts’ for the respondent to consider. Examples were similar enough that consumers see them as close substitutes, but dissimilar enough for them to clearly determine a preference. Each of these studies used the same overall design, so they could be compared to each other. This allowed for the research questions to be answered for an individual food category, a group of food categories, the entire set of food categories and cross-country comparisons.

### 2.3. Data Collection and Analysis

For the QMA study, no new topics emerged during the second focus group session, indicating that thematic saturation had been reached in the first focus group session and also indicating consistency between focus groups. After the QMA mapping, the participants generated groups of food categories that allowed for identification of linkages between different product and potential opportunity spaces to create new products. Data were transcribed from audio and video recordings by annotating important information given by participants and then used to identify recurring themes across sessions.

For the conjoint study, regression analysis of the ‘Premiumness’ classification was performed as described by Hughson [19]. The mathematical process illustrates how each element either adds to or detracts from the liking of a product as the effect of an element is based on its coefficient in the regression equation, with positive coefficients increasing acceptance and negative coefficients decreasing acceptance. The size of the coefficient reflects the strength of any effect. Those coefficients generated an equation similar to [18]: Premiumness classification = k0 + k1 (Element 1) + k2 (Element 2) +… k36 (Element 36). Where the constant, k0, is a theoretical value that functions as a correction factor and represents the likelihood that the respondent would like to consume a food concept that is not shown in the options. The element coefficients (k1–k36), represents the degree to which each level drives or reduces interest in a concept. Positive coefficients add to consumer interest while negative coefficients detract from interest. In total, there were 36 levels, with 6 within each of the 6 attributes. Thus, element also represents the frequency of selection for an individual level, within an attribute.

Methodology of [21] was applied to achieve segmentation of consumers into laggard (1–2), mainstream (3–4) and lead users (5–7). Segments were split by classifying the responses to the four questions shown in Table S4, into these three ranges and average of the regression coefficients across all levels for an attribute were calculated. This segmentation was determined through ‘self-identification’ and allowed an understanding of the predominant type of consumers in the given population, permitting targeting of products to a specify cohort. Percentage of attribute relative importance per segment was measured using the following equation which provided a way to represent how important an attribute was in a given segment relative to the other attributes.

$$\%=\frac{\sum \mathrm{absolute}\;\mathrm{average}\;\mathrm{of}\;\mathrm{regression}\;\mathrm{coefficients}\;\mathrm{per}\;\mathrm{attribute}}{\mathrm{Total}\;\sum \mathrm{of}\;\mathrm{regression}\;\mathrm{coefficients}\;\mathrm{for}\;\mathrm{all}\;\mathrm{attributes}}$$

## 3. Results

### 3.1. QMA Study

#### 3.1.1. Table of Meal/Snacking Behaviour

Participants provided detailed verbal descriptions of what they eat throughout the day. As shown in Table 1, Australians declared that they start eating around mid-morning (~11:00 a.m.) with a light snack, continue to snack across the day and consume the biggest portion of food in the afternoon (~4:00 p.m.). Often, they eat just for habit or a treat, not necessarily due to hunger. They rarely eat outside which is also true for Chinese. Most foods are ready-to-eat and consumed with their partner or accompanied with a beverage. Most foods consumed are shelf stable and the key protein source tends to be dairy oriented, e.g., cheese with biscuits.

The eating behaviour of the Chinese participants is totally different (Table 1) and almost the opposite to Australians. They have a healthier approach to food consumption as they are eating 3 regular meals during the day and snacking occasionally. This constitutes a habit along with warm water consumption as part of the traditional customs of their culture. For them, eating starts around early morning (~7:00 a.m.) and finishes by dinner (~6:00 p.m.) or after dinner (~8:00 p.m.).

#### 3.1.2. Meat Products Tasting

The Australians and Chinese description of their eating experience of the meat products presented is shown in Table 2. Positive and negative attributes for each product were recorded by each participant in the questionnaire. After completion of questionnaires and tasting, the facilitator allowed open discussion. Beef jerky and meat sticks were recorded as being ‘too hard’ in texture. In contrast to initial assumptions, most products were described ‘salty’. Surprisingly, even the skin of cocktail sausages and the fat layer of prosciutto were recorded as being difficult to eat. Meat floss was a very unfamiliar and undesirable product for the Australian participants.

Both Chinese and Australians described the beef jerky as the only ‘Beefy’ product, the liver pate had an unappealing appearance and too much pepper, the meat bolognese as easy to eat’, prosciutto as salty and meat sticks had a hard skin to bite through. In spite of this, Chinese had a different response to the meat products compared to Australians. For instance, Chinese did not have difficulty eating the skin of the cocktail sausages and even enjoyed the chewiness of the beef jerky. They were happy to take the time to slowly chew on it, which the Australians described as frustrating. Apart from liver pate, all products were described as having umami flavour by the Chinese participants, which seemed to be important for these consumers. Additionally, relevant was how convenient and/or easy the product was to carry around in a handbag. Prosciutto, apart from being unfamiliar, was also rejected for being ‘cured’, which was considered unhealthy by the Chinese group.

#### 3.1.3. QMA Perceptual Mapping

**Figure 2:**
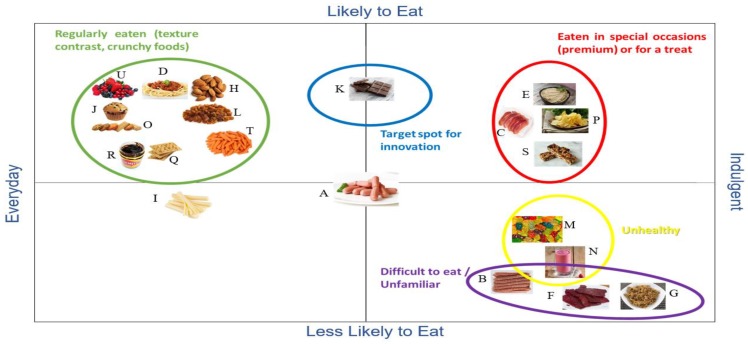
Perceptual map generated by Australian consumers in the qualitative multivariate analysis (QMA) discussions. The cocktail sausage image was the first shown, and everything else was mapped relative to it. (**A**) Cocktail sausages; (**B**) Meat sticks; (**C**) Prosciutto; (**D**) Meat bolognese; (**E**) Liver pate; (**F**) Chinese beef jerky; (**G**) Meat floss; (**H**) Almonds; (**I**) Cheese sticks; (**J**) Muffin; (**K**) Chocolate; (**L**) Raisins; (**M**) Gummies; (**N**) Smoothie; (**O**) Sweet biscuits; (**P**) Potato crisps; (**Q**) Crackers; (**R**) Vegemite; (**S**) Granola bar; (**T**) Baby carrots; (**U**) Mixed berries. The blue circle is drawn around the “target spot for innovation” and the foods in this target category would be frequently eaten with the desired texture contrast that can be consumed solo or shared with others. The green, red, yellow and purple shapes show groupings of products as indicated by the corresponding coloured text.

Insights regarding the snacks, for Australians are given in Figure 2. For instance, liver pate, prosciutto and potato chips were ranked as indulgent and easy to eat, except for the fat layers of the prosciutto which were removed before chewing. Dark chocolate was considered as ‘likely to eat’ and more indulgent for some than others, so it fell into two quadrants: Likely to Eat—Everyday and Likely to Eat—Indulgent. Cheese sticks were placed in the Everyday—Less Likely to Eat quadrant due to the negative perception the participants had of processed foods (even though consumers are eating cheese, they eat block cheese). Gummies and fruit smoothie were placed in the Less Likely to Eat—Indulgent as they were considered ‘unhealthy’ due to the perceived high amount of sugar.

**Figure 3:**
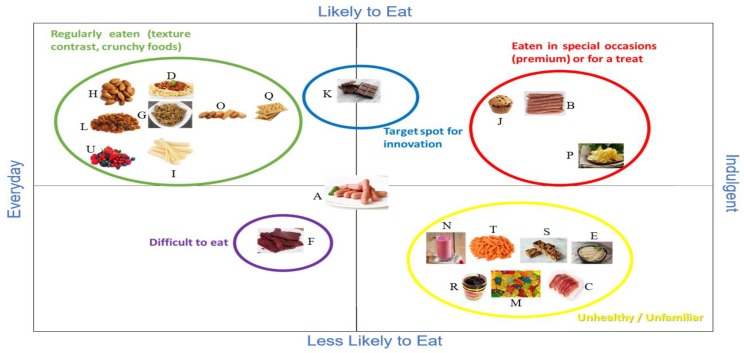
Perceptual map generated by Chinese consumers in the qualitative multivariate analysis (QMA) discussions. The cocktail sausage image was the first shown, and everything else was mapped relative to it. (**A**) Cocktail sausages; (**B**) Meat sticks; (**C**) Prosciutto; (**D**) Meat bolognese; (**E**) Liver pate; (**F**) Chinese beef jerky; (**G**) Meat floss; (**H**) Almonds; (**I**) Cheese sticks; (**J**) Muffin; (**K**) Chocolate; (**L**) Raisins; (**M**) Gummies; (**N**) Smoothie; (**O**) Sweet biscuits; (**P**) Potato crisps; (**Q**) Crackers; (**R**) Vegemite; (**S**) Granola bar; (**T**) Baby carrots; (**U**) Mixed berries. The blue circle is drawn around the “target spot for innovation” and the foods in this target category would be frequently eaten with the desired texture contrast that can be consumed solo or shared with others. The green, red, yellow and purple shapes show groupings of products as indicated by the corresponding coloured text.

At this stage, participants pointed out their negative attitude towards snacking, despite the fact that they snack throughout the day. This indicates that they are aware they are snacking and are also aware it is not a healthy daily eating habit. The ranking of snacks by the Chinese participants confirmed that they take a different approach to foods than Australians. Meat bolognese, berries, crackers, almonds, sultanas, potato chips, chocolate and gummies were the only foods that share the same location as for Australians, in the QMA map (Figure 3). It was interesting to find that dark chocolate was placed almost in the exact same spot by both groups. Beef jerky was placed in the Everyday—Less Likely to Eat quadrant due to the difficulty in eating because of its hard texture. Gummies and muesli bars were classified as ‘unhealthy’ mainly for the great amount of sugar some of those products have and placed in the Less Likely to Eat—Indulgent quadrant.

### 3.2. Conjoint Study

#### 3.2.1. Segments Classification into Laggard, Mainstream and Lead Users of Beef, Pork, Cheese and Chocolate Traits for Australians and Chinese Older Consumers

**Figure 4:**
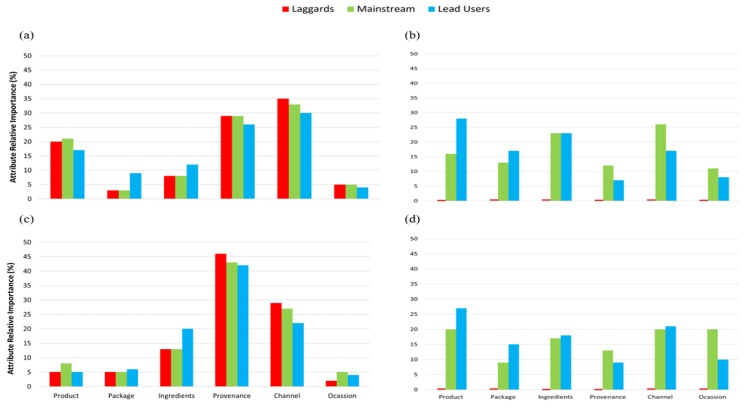
Average of the regression coefficients across all levels for an attribute for the three groupings of consumers being laggards (last to adopt a product), mainstream (general public) and lead users (fist to adopt a product) across the six attributes (product, package, ingredients, provenance, channel and occasion) for (**a**) beef Australia (*n* = 160, age = 55–84), (**b**) beef China (*n* = 32, age = 55–85), (**c**) pork Australia (*n* = 142 age = 55–84), (**d**) pork China (*n* = 36, age = 55–67). Regression coefficients of levels within attributes, within age groups and country, are given for selected food categories in Table 3 (for beef) and Table 4 (for pork).

**Figure 5:**
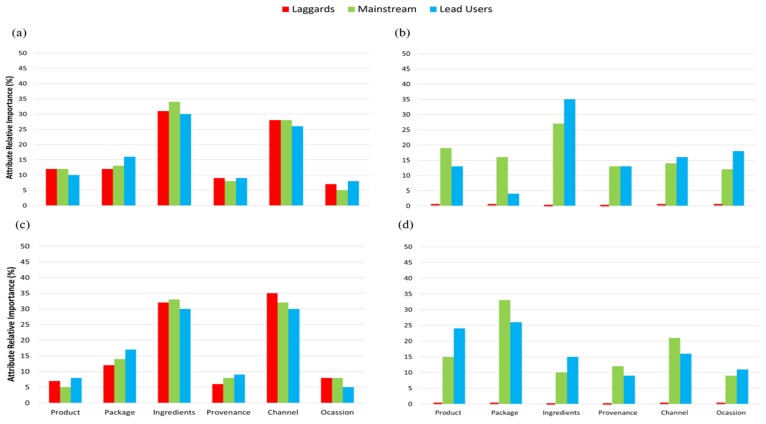
Average of the regression coefficients across all levels for an attribute for the three groupings of consumers being laggards (last to adopt a product), mainstream (general public) and lead users (fist to adopt a product) across the six attributes (product, package, ingredients, provenance, channel and occasion) for (**a**) cheese Australia (*n* = 134, age=55–80), (**b**) cheese China (*n* = 31, age = 55–80), (**c**) chocolate Australia (*n* = 144, age = 55–90) and (**d**) chocolate China (*n* = 29, age = 55–67). Regression coefficients of levels within attributes, within age groups and country, are given for selected food categories in Table 5 (for cheese) and Table 6 (for chocolate).

As shown in Figure 4 and Figure 5, from the segment grouping, it was evident that the importance of each trait (product, package, ingredient, provenance, channel and occasion) varies depending on country of birth. For instance, for Australians, provenance and channel were the most important traits within beef as for Chinese, product, ingredients and channel seems to be more relevant. A similar trend was observed for pork (Figure 4). For cheese, Australians consumers care for ingredients and channel, whereas Chinese mainly for ingredients. For Australians, ingredients and channel were most important for chocolate and for Chinese, it was packaging (Figure 5). For all food categories, it was found that Chinese consumers tend to act more as mainstream and lead users in contrast to Australian consumers where there are also a great part of the population acting as laggards.

#### 3.2.2. Utility Weight (Regression Coefficients) over Age and Gender for Most Important Beef, Pork, Cheese and Chocolate Traits for Australians and Chinese Consumers

It was interesting to look at how the importance of concepts for each food changes over age, gender and country. A subset table with the highest coefficients, i.e., >1, for each country is presented in this section (full-detailed tables for each food category can be found in Table S5). For beef, concepts for product and ingredient uniqueness, along with provenance and channel were the most relevant for both groups of consumers. As shown in Table 3, it is not surprising that in China, the concept of stir fry beef is more acceptable than in Australia for both male and female. However, a tender and juicy piece of beef sirloin was very appealing, especially for Australians. Preferences for beef ingredients differed between countries. Chinese preferred lean meat but flavoursome, and for Australians, a premium meat was better. For provenance, both groups liked an environmentally friendly meat production. Traceability was also important for Australians but not so much for Chinese. As for the place to buy beef, Australians picked “local store” and “supermarket” and Chinese mainly the later.

For pork, product, provenance and channel were important (Table 4). Interestingly, in product uniqueness, the concept of “authentic, tender and juicy Australian pork loin chop” was more appealing for both groups than just plain “pork loin chop”. Important to note that the word ‘authentic’ made a massive difference for liking or not this food. Provenance concepts of pork mattered more for Australian consumers. Pork packaged and prepared in Australia and produced taking into consideration animal welfare and environmentally friendly practices, were most preferred. As for channel, Australians preferred to buy pork from a small market/specialty store, especially as people aged. In contrast, Chinese preferred to buy pork from a local seller.

In the cheese category, ingredient uniqueness and channel were important for both countries as shown in Table 5. It was interesting to see that as Australian women age, they become more concerned about animal wellbeing as “made with milk from a single cow free to roam on green pastures” was scored higher (Table 5). In contrast, the concept of “made with fresh Australian milk” had a negative score which increased with age. Importantly, Australians would buy cheese in any food store whilst Chinese are more likely to buy it from an Australian seller.

For chocolate, the most important attributes were package, ingredient and channel for both countries (Table 6). Individual wrap and gift pack were most preferred. The concept of “Australian cocoa beans blended with all Australian ingredients for a premium chocolate” was highly appealing for Australian women as they age (Table 6). The concept of healthiness was also important when buying chocolate. These findings can help to understand why chocolate was placed in the “likely to eat” area of the QMA maps (Figure 2 and Figure 3) as it was thought to be a premium product and the majority of participants were women. As for channel, Australians would buy chocolate in any food store and Chinese mostly in the supermarket.

## 4. Discussion

Opportunities for the food industry in general, include new products for older consumers. Hence, it is important to understand the thinking/mind of older consumers to be able to meet their product requirements.

Healthy life expectancy (HALE) considers the time spent living with disease and injury and is described by the World Health Organization (WHO) as “the average number of years that a person can expect to live in “full health” by taking into account “years lived in less than full health due to disease and/or injury” [22] and it is well known that a poor diet can cause disease. The HALE for Australia was 83.4 and for China 76.9 years for 2019 [23].

### 4.1. QMA Study

A snack is defined as foods that can be eaten in place of, or in between meals, that are convenient because they can be quick and easy to eat [24]. Snacking (‘snackification’) is one of the top five consumer trends in 2019 and is expected to gain further momentum in the future [25]. Snacking now makes up nearly half of all eating occasions and is one of the most profound changes in consumers’ behaviour. Time-poor consumers, rising health consciousness, higher discretionary incomes and demand from grocery are drivers of snacking [26]. Snacking habits are no longer purely the domain of children and people under 30´s, with 96% of Australians consuming some sort of snack on a regular basis [8]. Thus, snacking has now pervaded all segments of society, including ‘seniors’. About 84% of non-institutionalised adults aged 65 years and older snack [27]. Snacking on foods and beverages between meals can be an effective way to increase daily calories in older adults [27]. Older adult non-snackers consumed 134 fewer kcal than the 1600 kcal recommended by the US Department of Agriculture [28] for sedentary older women, while snackers consumed 1718 kcal daily [5]. Food intake in general is affected as people get older since texture and flavour of foods, particularly meat, is perceived differently. Products such as beef jerky, that once were easy to eat can become difficult, as people age. It is also believed that taste bud sensitivity might decrease with age, causing reduced perception of certain flavours, such as saltiness.

The qualitative data collected on snacking behaviour shows that older Australian consumers are eating small portions of food throughout the day in place of meals. Mostly, they are consuming ready-to-eat products. Baby boomers (people born between 1946 and 1965) snack to avoid consuming larger meals, often alone or just in a group of two [26]. This was not true for older Chinese who eat regular meals throughout the day.

Although the recommended daily intake for adults is 0.8 g protein/kg body weight, some aged care nutritionists have recommended that older people should increase their daily intake of protein to 1.2 g per body weight [29]. For example, a person weighing 70 kg will need to increase their intake of protein from 56 to 84 g per day. An Australian Health Survey in 2011–2012 showed that older males are only getting 1.6 servings of lean meat and poultry, fish, eggs, tofu, nuts and seeds, and legumes/beans vs. the recommended 2.5 servings while older women are getting only 1 serving vs. the recommended 2 servings [30]. Protein intake should be increased in older adults, given its importance in delaying sarcopenia. Snacking or eating protein dense foods, such as meat products, can create a positive impact in increasing protein intake.

When considering the importance of texture vs. flavour, the assumption that older adults have difficulty eating beef jerky and meat sticks due to their dry and hard nature [31,32] turned out to be confirmed by the Australian participants. Many of them had consumed these products at a younger age and were now missing the textures and their flavours. Interestingly, eating the skin of cocktail sausages was also a difficult task for most of the Australian participants. In contrast, Chinese participants enjoyed the chewiness of beef jerky and ate the cocktail sausages without peeling off the skin. It is clear that while age can have an effect on eating quality of meat, there is also a cultural or geographical overlay.

Age-related changes in taste and chemosensory acuity may be the result of impaired swallowing and difficulty chewing, because of associated problems with teeth and gums and potential issues arising from the need to take medications for various health conditions [31]. This needs to be considered when developing food products for older adults. In our study, no participants were taking strong medication, hence this was not relevant. In a relevant study, authors fed subjects beef with contrasting textures (either tough and dry or tender and juicy, obtained by varying post-mortem aging and cooking temperature) [32]. Regardless of the beef texture, chewing duration before swallowing was longer, and bolus residual strength was greater for older compared to younger adults. Although there have been several studies developing meat products for the elderly [33,34,35,36], there is still insufficient information on a wide range of meat products that are easy to eat and consequently increase protein consumption in older people. These overall difficulties with texture, drives older consumers to consider more softer textures in their food choices and avoid eating meat. If consumption of meat and meat products is reduced, significant nutritional advantages are lost, and total intake of energy and high-quality protein may fall below dietary requirements [33].

Understanding what the appropriate texture range is for a product becomes critical to creating a novel red meat snack that will be easily consumed, while also providing the satisfaction of meat consumption. When eating meat products, particle size was found to be important for ease of break down in the mouth. For instance, in the present study, fine particles in liver pate were preferred by older Australians over ‘fibrous’ pieces of meat bolognese as the term ‘fibrous’ can include ‘stringiness’, which can make the product difficult to eat. A soft texture with a ‘little bit of bite’, ‘melty’ but ’not fibrous mouthfeel’ and ’moisture’, during consumption for Australians and in the product itself for Chinese, were also important traits for older consumers.

As we age, the way our senses (hearing, vision, taste, smell, touch) function changes and gustatory dysfunction may indeed be related to the normal ageing process [37]. Participants, especially older Australians, found cocktail sausages too salty. Again, this may present a gap in the marketplace for older consumers, as they are perceiving a salty flavour, which goes against their desire for low-salt products for health reasons.

In the QMA mapping and group discussion, the moderator explored how participants felt towards processed foods and what they considered as ‘unhealthy’. Interestingly, older Australians defined ‘unhealthy’ as a high content of fat, salt and sugar, which agrees with the global definition [24]. However, for older Chinese, a high amount of sugar seemed to be the biggest reported trigger for unhealthiness, with less focus on fat and salt. Both groups had a negative perception of processed foods, which they classified as too artificial, e.g., cheese sticks vs. cheese block. The latter product is preferred as it is considered closer to a whole food, and less processed.

### 4.2. Conjoint Study

Country of birth was a defining factor for classifying importance of each product trait as shown in the segmentation results (Figure 4 and Figure 5). Ethnicity is often found to be important for food perception and consumption, possibly due to differences in oral physiology and anatomy between consumers belonging to different ethnicities, which might cause differences in food oral processing behaviour [38,39]. Others have also found ethnicity to be important when investigating the consumer perception of meat alternatives in USA, India and China [40].

Laggards are the last users to adopt a product and generally they prefer simple products [41]. They are the last to pick up on innovations and only buy an innovative product when it works completely flawlessly, and all traditional alternatives are no longer available [42]. Mainstream users, as the name implies, refers to the general public or majority of consumers [43]. They rely on security, trying the products after they have been proven and the system “forced them to” [42]. Lead users are users whose present strong needs will become general in a marketplace months or years in the future [21]. Lead users are defined as open to innovation and pioneers among all users in a population. They actively participate in product development, often creating their own prototypes based on their need [42].

Older Chinese consumers seemed to be more ‘adventurous’ when choosing food products compared to older Australian consumers who behave more as mainstream and laggards. In contrast, the older Australian QMA participants showed less interest in trying new products unless they were highly recommended by trusted people. Chinese culture differs significantly from Western and even other Asian cultures, so consumers have different values and a different perception of product attributes [44,45,46].

Product and ingredient uniqueness, provenance and channel concepts of beef were important in terms of premiumness for both older Chinese and Australians consumers. However, the importance of each level within attributes differed between Australia and China. According to [47], Chinese consumers may desire similar product features (e.g., brand name, quality and flavour) to Western consumers, but the value that consumers attach to the same product may differ cross-nationally. For instance, whole meat cuts and premium meat are highly preferable for Australians in comparison with Chinese.

Product uniqueness, provenance and channel concepts were the most relevant attributes for premiumness for both older Australians and Chinese when buying pork. Chinese like to buy their pork form a Chinese seller, but if the meat is from Australia, their interest increased. The same cannot be said for Australians who said they preferred to by pork from a specialty store (e.g., local butcher). In a study investigating consumer preference for pork in different Asian countries, including China, specific consumer preferences differed for meat cut choices, provenance, price and sensory characteristics, and the authors suggested this should be considered carefully to increase product consumption [48] by offering the specific products each cohort is demanding.

For cheese, ingredient and channel were important for both countries. Using Australian milk for cheese manufacture was polarising for Australians and Chinese, as were other elements. This might be due to the low cheese consumption by older Chinese consumers. Furthermore, older Chinese preferred dry dairy products, including those from Australian origin [49], which is reflected in our findings.

For chocolate, channel was important for both countries. “Bush ingredients”, which implies native Australian or natural food, was defined as important for respondents in the conjoint study and agrees with the finding from the QMA of participants wanting to consume less processed cheese, as ingredients play a key role for a product to be considered as more or less processed. In a study on consumer views on “healthier” processed meat, authors reported findings from seven focus groups where participants considered that, in order to improve the (perceived) healthiness of those products, the focus should be on the use of better-quality ingredients, e.g., natural ingredients, and less salt, fat, preservatives and other additives [50].

## 5. Conclusions

This research revealed that older Chinese are eating in a healthier way than older Australians. It was shown that older Chinese are eating three regular meals during the day and not snacking much, whilst Australians are doing the opposite. In the older population, snacking appeared to be more important for Australians than Chinese. Both Australians and Chinese reported that they avoid overly processed and sugary foods. These findings demonstrated that older adults, especially Australians, need more healthy choices for snacks, that fit their needs for the proper texture and familiar flavour. A marked difference was observed among participants depending on their country of origin. These results were reinforced with the findings from the conjoint analysis which supported the importance of demographics for perception and preference of, in this case, premium foods. For older Chinese, one possibility might be to create a product, with similar characteristics to meat floss, that could be eaten as a stand-alone product, or used as filling/ingredient in other dishes. Clearly, there is opportunity to create a nutrient dense product to better fit their need to a larger range of healthy snack foods. Our research suggest that the novel product should be ‘ready to eat’ and bite-sized, to align with the snacking behaviour of older people. The ability to describe the product space characteristics and how it fits with the behaviours, health rules and emotions of older consumers provides a starting point for the meat industry to create a differentiated snack that will fit their lifestyles. For the product to be successful, it is important to consider demographics as a factor that is important for food choices. However, older adults in general are not a homogeneous group. Depending on the age range and other factors, they have different nutritional needs. Further research with specific older age subgroups is needed to determine the most effective approaches for promoting health and wellbeing.

## Figures and Tables

**Table 1:** Key insights from the discussion during the construction of the meal/snacking behaviour table (Stage 1) of older Australian and Chinese adults.

Time of Consumption	Australian *	Chinese **
Early morning	No food consumed	Consumption of warm water first thing in the morning
Breakfast	Only tea or orange juice	Consumption of proper breakfast, lunch and dinner across the day for health, habit, hunger or share with family and friends
Mid-morning	A dry/crunchy food with coffee, consumed with partner	
Lunch	A dry/crunchy food, combination of sweet and salty, eaten at home, solo or with partner	
Afternoon	Most food consumption (cheese, biscuits, fruit/vegetables and sweets) due to hunger or habit with coffee or tea, eaten solo or with partner	Mid-morning and afternoon snacks only if hungry but unlikely
Dinner	No ‘proper’ dinner is consumed. Consumption of something sweet or crunchy for a treat	
After dinner	The trend is to consume mostly sweet, crunchy foods for a treat, boredom or habit. Eaten at home with a hot beverage (coffee, tea, hot chocolate)	Snack for gut health or sleep better. Mainly dairy-based, i.e., plain yogurt, milk
Other comments	Do not cook very often, prefer ready-to-eat/convenient foods	No liquids are consumed during meals, just before or after, still cook by themselves, enjoy homemade and fresh food, warm water is consumed before bed

* *n* = 16; two sessions: *n* = 8 in each. ** *n* = 21; two sessions: *n* = 12 and 9 respectively.

**Table 2:** Results from the tasting of meat products (Stage 2) showing summary of responses from older Australian and Chinese adults, replicated across sessions in the qualitative multivariate analysis (QMA). The products with the softest texture are given at the top of the table and hardest at the bottom of the table.

Meat Product	Australian (*n* = 16)	Chinese (*n* = 21)
Liver pate	Spreadable, special occasions, too peppery, uninviting appearance (familiar)	Too soft, too peppery, uninviting appearance (not familiar)
Meat floss	Disgusting, hard to eat in terms of familiarity, ‘fibrous’ (not familiar)	Delicious, easy to eat, crispy and soft, umami (familiar)
Meat bolognese	Appetising, easy to eat, bland (familiar)	Meaty, moist, soft, easy to eat, umami (familiar)
Cocktail sausage	Party food, easy to eat filling, skin too hard (familiar)	Meaty, soft, easy to eat, too processed, umami (familiar)
Prosciutto	Appetising, snack food, fatty, chewy, salty (familiar)	Convenient, nutritious, too salty, not tasty, umami (not familiar)
Beef jerky	‘Beefy’ (not familiar)	‘Beefy’, meaty, hard to eat, chewy, umami (familiar)
Meat sticks	Party food, soft filling, hard skin, flavourless, difficult to bite (familiar)	Meaty, tasty, convenient, dry, tough skin, non-nutritious, umami (familiar)

**Table 3:** Premiumness classification ^1^ (see footnote for equation) given to each level (1, 2), within each attribute (product, ingredient, provenance, channel) which makes an element for beef with the data divided for country (Australia, China), age group (25–44, 45–54, ≥55), sex (male, female). A high positive value indicates the consumer considered the element to be more premium within the attribute compared to other options and conversely negative values indicate less premiumness.

	Country:	AUSTRALIA						CHINA					
	Gender:	Male			Female			Male			Female		
	Age Group:	25–44	45–54	≥55	25–44	45–54	≥55	25–44	45–54	≥55	25–44	45–54	≥55
	Number of Respondents:	48	40	89	92	58	71	131	41	15	151	44	17
Attribute	Element												
**PRODUCT**	Beef stir fry, cut in just the right size	0.04	0.18	−0.20	−0.11	0.16	0.07	0.40	0.12	1.17	0.11	0.28	0.64
	Beef sirloin, tender and juicy every time	2.78	2.59	2.15	2.27	2.53	2.44	0.83	0.62	0.38	0.67	0.62	0.69
**INGREDIENT**	Lean heart healthy beef, raised to have monosaturated fats to lower your blood pressure and cholesterol, but still have lots of flavour	−0.59	−0.46	−0.50	−0.28	−0.66	−0.55	0.34	0.57	0.71	0.58	0.71	0.72
	Premium pasture fed beef from Blackmore’s Wagyu, Cape Grim, or Minderoo	0.84	0.52	0.31	0.54	0.77	0.23	−0.23	−0.18	−0.26	−0.15	−0.20	−0.67
**PROVENANCE**	Authenticated, traceable back to the farm	0.74	0.96	1.10	0.64	1.00	0.98	−0.01	0.41	−0.03	0.15	0.17	0.14
	Raised on a small family farm, grass feed using biodiverse pastures, hormone free, using sustainable farming practices	1.50	1.36	1.58	1.61	1.74	1.99	0.33	0.20	0.11	0.39	0.44	0.10
**CHANNEL**	Available at my local store	1.45	1.43	1.75	1.69	1.50	1.86	0.10	0.03	0.96	−0.01	0.00	−0.14
	Available in the supermarket	1.34	1.33	1.57	1.37	1.62	1.83	0.59	0.40	1.01	0.67	0.71	0.34

^1^ Premiumness classification = k0 + k1 (Element 1) + k2 (Element 2) + … k36 (Element 36). Where k0: constant and k1–k36 elements are concepts listed above within attributes. k0–k36 are the partial coefficients which are calculated in the equation and add up to zero.

**Table 4:** Premiumness classification ^1^ (see footnote for equation) given to each level (1, 2), within each attribute (product, provenance, channel) which makes an element for pork with the data divided for country (Australia, China), age group (25–44, 45–54, ≥55), sex (male, female). A high positive value indicates the consumer considered the element to be more premium within the attribute compared to other options and conversely negative values indicate less premiumness.

	Country:	AUSTRALIA						CHINA					
	Gender:	Male			Female			Male			Female		
	Age Group:	25–44	45–54	≥55	25–44	45–54	≥55	25–44	45–54	≥55	25–44	45–54	≥55
	Number of Respondents:	55	41	86	83	66	56	157	39	25	122	50	11
Attribute:	Levels:												
**PRODUCT**	Pork loin chop	−0.46	−0.64	−0.70	−0.58	−0.53	−0.47	−0.46	−0.18	−0.69	−0.38	−0.63	−0.19
	Authentic, tender and juicy Australian pork loin chop	0.47	0.10	0.01	0.13	0.17	0.07	1.25	0.79	1.10	1.16	1.50	1.10
**PROVENANCE**	Australian meat packaged and prepared in Australia	1.05	1.19	1.02	0.87	0.82	1.37	0.15	−0.01	0.39	0.08	−0.02	−0.18
	Raised on a small family farm, grass feed using biodiverse pastures, hormone free, using sustainable farming practices	2.05	2.01	2.20	2.39	2.07	2.24	0.12	0.15	0.32	0.15	−0.01	−0.11
**CHANNEL**	Available at a small market/specialty store	0.77	1.02	1.21	0.75	0.64	1.31	−0.16	−0.51	0.21	−0.51	−0.51	−0.83
	Available online from a Chinese seller	0.11	0.04	−0.12	0.15	0.22	−0.39	0.78	1.05	0.84	0.91	0.76	1.05

^1^ Premiumness classification = k0 + k1 (Element 1) + k2 (Element 2) + … k36 (Element 36). Where k0: constant and k1–k36 elements are concepts listed above within attributes. k0–k36 are the partial coefficients which are calculated in the equation and add up to zero.

**Table 5:** Premiumness classification ^1^ (see footnote for equation) given to each level (1, 2), within each attribute (ingredient, channel) which makes an element for cheese with the data divided for country (Australia, China), age group (25–44, 45–54, ≥55), sex (male, female). A high positive value indicates the consumer considered the element to be more premium within the attribute compared to other options and conversely negative values indicate less premiumness.

	Country:	AUSTRALIA						CHINA					
	Gender:	Male			Female			Male			Female		
	Age group:	25–44	45–54	≥55	25–44	45–54	≥55	25–44	45–54	≥55	25–44	45–54	≥55
	Number of respondents:	46	46	62	92	65	72	146	32	14	164	28	17
Attribute:	Levels:												
**INGREDIENT**	Made with fresh Australian milk	1.31	1.54	2.12	1.57	1.99	2.15	−0.11	−0.55	−0.61	−0.18	0.11	−0.51
	Made with milk from a single cow free to roam on green pastures	0.87	1.06	1.15	1.09	0.97	1.48	0.51	0.89	1.23	0.41	0.50	0.86
**CHANNEL**	Available at all stores where food and beverages are sold	1.05	1.32	1.39	1.22	1.57	1.36	0.11	−0.21	−0.46	0.15	0.32	0.30
	Available online from an Australian seller	0.02	−0.05	−0.26	0.00	0.04	0.17	0.42	0.18	0.31	0.65	0.07	0.47

^1^ Premiumness classification = k0 + k1 (Element 1) + k2 (Element 2) + … k36 (Element 36). Where k0: constant and k1–k36 elements are concepts listed above within attributes. k0–k36 are the partial coefficients which are calculated in the equation and add up to zero.

**Table 6:** Premiumness classification ^1^ (see footnote for equation) given to each level (1, 2), within each attribute (package, ingredient, channel) which makes an element for chocolate with the data divided for country (Australia, China), age group (25–44, 45–54, ≥55), sex (male, female). A high positive value indicates the consumer considered the element to be more premium within the attribute compared to other options and conversely negative values indicate less premiumness.

	Country:	AUSTRALIA						CHINA					
	Gender:	Male			Female			Male			Female		
	Age Group:	25–44	45–54	≥55	25–44	45–54	≥55	25–44	45–54	≥55	25–44	45–54	≥55
	Number of Respondents:	136	44	66	155	49	78	144	40	15	143	40	14
Attribute:	Levels:												
**PACKAGE**	Individual wrap	0.42	0.80	0.56	0.64	0.68	0.68	0.52	0.68	0.42	0.64	0.59	0.80
	Gift pack	1.38	2.38	1.22	1.50	1.81	0.86	1.05	0.81	1.14	1.08	1.19	0.84
**INGREDIENT**	Made from wild bush grown cocoa beans that provide the maximum health benefits from antioxidants	0.52	0.20	0.56	0.72	0.68	0.73	0.31	0.45	0.49	0.35	0.27	0.42
	Australian cocoa beans blended with all Australian ingredients for a premium chocolate	2.01	2.03	2.35	2.14	2.22	2.74	0.14	0.20	0.13	0.33	−0.05	0.11
**CHANNEL**	Available at all stores where food and beverages are sold	1.07	1.06	1.30	1.04	1.18	1.26	0.05	0.21	0.03	0.16	0.00	0.36
	Available in the supermarket	0.92	1.34	1.34	1.02	1.48	1.50	0.57	0.33	0.69	0.57	0.61	0.35

^1^ Premiumness classification = k0 + k1 (Element 1) + k2 (Element 2) + … k36 (Element 36). Where k0: constant and k1–k36 elements are concepts listed above within attributes. k0–k36 are the partial coefficients which are calculated in the equation and add up to zero.

## Supplementary Materials

The following are available online at https://www.mdpi.com/2304-8158/9/4/426/s1, Table S1: Moderator guide used for group discussion in QMA. Table S2: Lexicon for sensory attributes of meat products [51]. Table S3: Conjoint design for product concepts per food category (36 levels). Table S4: Questions included for segments stratification, Table S5: Utility weight (regression coefficient) for beef, pork, cheese and chocolate concepts per attributes according to age, gender and country of origin (Australia or China), Figure S1: Example per food category of how the respondent saw the concepts in the survey, Table S5: Utility weight (regression coefficient) for concepts per food category and attributes according to age, gender and country of origin (Australia or China).
