# Supplementary material for: Exploring Meal and Snacking Behaviour of Older Adults in Australia and China
Source: Foods. 2020 Apr 3;9(4):426. doi: 10.3390/foods9040426 (PMC7231097; doi:10.3390/foods9040426)
Supplement: Supplementary file 1 [file foods-09-00426-s001.pdf]

Supplementary Materials:

Table S1: Moderator guide used for group discussion in qualitative multivariate analysis (QMA).

STAGE	DURATION	DESCRIPTION	OBJECTIVE	KEY QUESTIONS/POINTS
Introduction	10 minutes	• Researchers and project introduction<br>• Paperwork completion (consent form, PLS form)<br>• Honesty – no right or wrong answers	To meet the respondent and set the scene for the discussion	Snacks Definition: these are foods that can be eaten in place of, or in between meals, that are convenient because they can be quick and easy to eat. The term "snack food" does not only apply to some of the newer products such as potato crisps, but it also includes many traditional food items [24].
5Ws (when, what, why, where who with)	40 minutes	• Introduce table activity<br>• Ask for opinions<br>• Complete the table	Investigate why they are snacking (fuel, treat, boost)	Key Words to Describe Snacks:<br>• For Fuel: Nourish, Satisfy, Sustain<br>• For Treat: Delight, Distraction, Sensation<br>• For Boost: Mental, Refreshing
Snacks Tasting and Mapping	60 minutes	• Introduce mapping activity and usage of pictures to describe snacking preferences<br>• Evaluate one product at a time, allow everyone to participate	To measure snaking behaviour, needs and wants from food and meat products	• Cocktail sausage is our reference point for mapping. Map it first and place it in the middle.<br>• Track if they have a variation for fruit, veggie and cracker.<br>• When do you snack?<br>• How do you describe its texture (crunchy, springy, firm, grainy)?<br>• How do you describe its flavour (bland, tasty, sour, salty)?<br>• How do you describe its appearance (attractive, flat, lumpy)?<br>• Which product did you prefer?<br>• What make it better over the other types of snacks?<br>• Is healthiness an important factor for you to choose a snack? why?<br>• Is the protein content of a snack an important factor to you? why?

				• At what time of the day do you consume these products? why?<br>• Do you have difficulty eating any of these products? Which one? why?
Compensation	10 minutes	• Gift handover<br>• Gratitude expression	To compensate respondent for their support and time	

Table S2: Lexicon for sensory attributes of meat products. Derived from [51].

FLAVOUR	APPEARANCE	TEXTURE	AROMA
Bland	Off-putting	Dry	Rancid
Contrast	Pale	Simple	Fermented
Fatty	Crumbly	Chewy	Smoky
Sour	Dry	Crumbly	Fatty
Bitter	Fatty	Tough	Rotten
Rancid	Slimy	Lumpy	Rubbery
Hot	Lumpy	Mushy	Beefy
Spicy	Thick	Sticky	Fresh
Artificial	Soft	Thick	Cooked
Salty	Juicy	Hard to eat	Spicy
Sweet	Hot	Easy to eat	Meaty
Umami	Healthy	Tender	Umami
Fresh	Colourful	Springy	Other
Sharp	Appetising	Juicy	
Tasty	Other	Crispy	
No contrast		Complex	
Multi-taste		Soft	
Other		Other	

Table S3: Conjoint design for product concepts of **beef** (36 levels).

Category	Attribute 1 - Product uniqueness	Attribute 2 - Packaging uniqueness	Attribute 3 - Ingredient uniqueness	Attribute 4 - Provenance process	Attribute 5 - Channel	Attribute 6 - Occasion
**BEEF**<br>**Level 1**	Unique breeds like older Longhorns that have a chance to develop more flavour, with a delicate beefy flavour and a slightly acid finish without having a very high fat content	Map tray sealed on top clear with loin cut 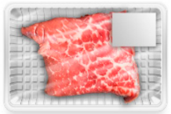	Fresh Australian beef	Australian meat packaged and prepared in China	Available at all stores where food and beverages are sold	Something I would have by myself at home
**Level 2**	Beef stir fry, cut in just the right size	Map tray sealed on top black with loin cut 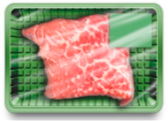	Certified organic Australian beef	Australian meat packaged and prepared in Australia	Available at my local store	Something I would share with friends and family
**Level 3**	Slow cooked beef with onion and gravy, just heat and eat	Map tray seal with shiny black tray 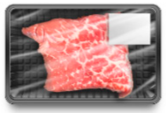	Traditional Australian Breeds like Brahaman or Angus	Authenticated, traceable back to the farm	Available in the supermarket	Something I would share when I am out with friends and family
**Level 4**	Cuts like heart, liver, kidney… full of nutrition	Darfresh Vacuum sealed, green tray 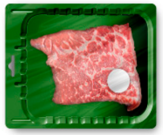	Unique breeds like older Longhorns that have a chance to develop more flavour, with a delicate beefy flavour and a slightly acid finish without having a very high fat content	Aged using Traditional craftsmanship practices like dry aging for 35 days to tenderize and create a distinctive melt in your mouth flavour	Available at a small market / specialty store	Something I would have for a special occasion like a festival or holiday

**Level 5**	Classic Beef Roast cut and tied, ready to cook	Matt black flat tray with tiny lip Darfresh vacuum, meat higher than lip with gold base 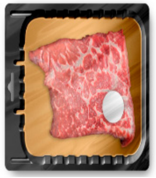	Lean heart healthy beef, raised to have monosaturated fats to lower your blood pressure and cholesterol, but still have lots of flavour	Raised on a small family farm, grass feed using biodiverse pastures, hormone free, using sustainable farming practices	Available online from a Chinese seller	Something I would have for a special celebration
**Level 6**	Beef sirloin, tender and juicy every time	Butcher board square wood with meat on top 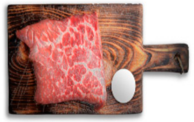	Premium pasture fed beef from Blackmore's Wagyu, Cape Grim, or Minderoo	Highest quality premium meat, recommended by celebrities, and chefs as their favourite	Available online from an Australian seller	Something I would have/give to my partner to show how much I care

Table S3 cont.: Conjoint design for product concepts of **pork** (36 levels).

Category	Attribute 1 - Product uniqueness	Attribute 2 - Packaging uniqueness	Attribute 3 - Ingredient uniqueness	Attribute 4 - Provenance process	Attribute 5 - Channel	Attribute 6 - Occasion
**PORK** **Level 1**	Pork loin chop	Map tray sealed on top clear with loin cut 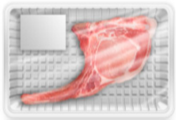	Australian pork	Australian pork packaged and prepared in China	Available at all stores where food and beverages are sold	Something I would have by myself at home
**Level 2**	Australian pork loin chop	Map tray sealed on top green with loin cut 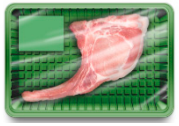	Fresh Australian pork, lean and heart smart	Australian pork packaged and prepared in Australia	Available at my local store	Something I would share with friends and family
**Level 3**	Fresh Australian pork loin chop	Ma [illegible] shiny black tray 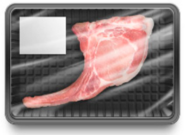	Healthy, natural Australian pork	Fresh quality pork, 100% Australian farmed and butchered	Available online with little information on country of origin	Something I would share when I am out with friends and family
**Level 4**	Tender and juicy Australian pork loin chop	Darfresh vacuum sealed, green tray 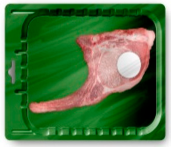	Certified free range Australian pork	Traditional European Berkshire breed. Farmed in Australia	Available at my Supermarket	Something I would have for a special occasion like a festival or holiday

**Level 5**	Authentic Australian pork loin chop	Matt black flat tray with tiny lip Darfresh vacuum, meat higher than lip with gold base 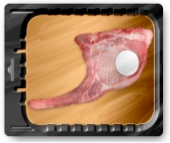	Locally sourced, tasty Australian pork	Australian pork raised on a small family farm, hormone free, using sustainable farming practices	Available online, fully authenticated, traceable back to local farm	Something I would have for a special celebration
**Level 6**	Authentic, tender and juicy Australian pork loin chop	Butcher board square wood with meat on top 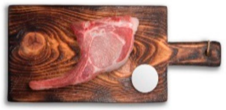	Traditional Australian pork farmed using sustainable practices	Highest quality Australian premium pork, recommended by butchers and chefs as their favourite	Only available from select butchers and markets	Something I would have/give to my partner to show how much I care

Table S3 cont.: Conjoint design for product concepts of **cheese** (36 levels).

Category	Attribute 1 - Product uniqueness	Attribute 2 - Packaging uniqueness	Attribute 3 - Ingredient uniqueness	Attribute 4 - Provenance process	Attribute 5 - Channel	Attribute 6 - Occasion
**CHEESE** **Level 1**	Premium quality	Slice cheese plastic individual wrap 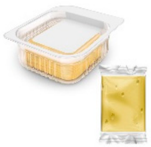	Made with fresh Australian milk	Prepared in a modern clean facility	Available at all stores where food and beverages are sold	Something I would have by myself at home
**Level 2**	Certified organic	Cheese sticks 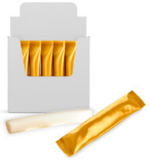	Made with fresh Chinese milk	Authenticated, traceable back to the dairy farm	Available at my local store	Something I would share with friends and family
**Level 3**	Flavourful cheese	Laughing cow triangle foil wrap in wheel 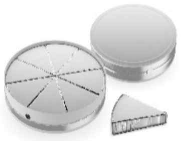	Made with Australian bush ingredients to enhance health and wellbeing	Hand crafted by a chef with a unique recipe	Available in the supermarket	Something I would share when I am out with friends and family
**Level 4**	Lightly flavoured cheese	Baby bell wax peel 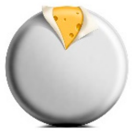	Made with fresh Australian A2 high protein milk	Handcrafted using traditional methods	Available at a small market / specialty store	Something I would have for a special occasion like a festival or holiday

**Level 5**	Cheese that is full of pieces of fruit or nuts	Tray platter with map packaging like catering for sharing 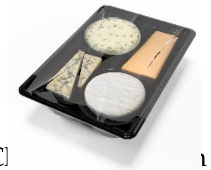	Made with fresh milk, for the best tradition can offer	Made with an uncompromising attitude to design and creation from years of expert knowledge	Available online from a Chinese seller	Something I would have for a special celebration
**Level 6**	A low lactose cheese that is delicious and easy on the tummy	Cl[illegible]n DarFresh vacuum pack 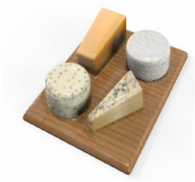	Made with milk from a single cow free to roam on green pastures	Limited small batches, prepared at the dairy, when all the ingredients are in peak season, quickly processed for a unique special flavour	Available online from an Australian seller	Something I would have/give to my partner to show how much I care

Table S3 cont.: Conjoint design for product concepts of **chocolate** (36 levels).

Category	Attribute 1 - Product uniqueness	Attribute 2 - Packaging uniqueness	Attribute 3 - Ingredient uniqueness	Attribute 4 - Provenance process	Attribute 5 - Channel	Attribute 6 - Occasion
**CHOCOLATE**<br><br>**Level 1**	Premium quality	Silver wrap 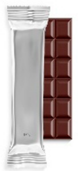	Cocoa from around the world is combined with Australian milk & sugar	Prepared in a modern clean facility	Available at all stores where food and beverages are sold	Something I would have by myself at home
**Level 2**	Certified organic	Green wrap 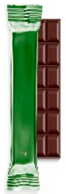	Cocoa from around the world is combined with Chinese milk & sugar	Authenticated, traceable back to farms where the ingredients came from	Available at my local store	Something I would share with friends and family
**Level 3**	Full bodied dark chocolate	Pouch 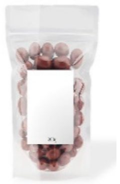	Made from wild bush grown cocoa beans that provide the maximum health benefits from antioxidants	Hand crafted by a chocolatier, with a unique recipe	Available in the supermarket	Something I would share when I am out with friends and family
**Level 4**	Smooth plain slightly sweet milk chocolate	Blister pack 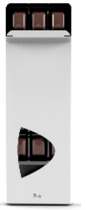	Globally sourced cocoa blended with just a touch of sugar for a bitter chocolate, full of antioxidants	Handcrafted using traditional methods	Available at a small market / specialty store	Something I would have for a special occasion like a festival or holiday

**Level 5**	Full of chewy, crunch nuts with the chocolate	Individual wrap 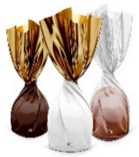	Single origin globally sourced cocoa beans	Made with an uncompromising attitude to design and creation from years of expert knowledge	Available online from a Chinese seller	Something I would have for a special celebration
**Level 6**	Filled with a soft smooth filling	Gift pack 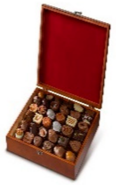	Australian cocoa beans blended with all Australian ingredients for a premium chocolate	Limited rare edition, cocoa beans are picked and roasted in 24 hours from the moment of peak timing once a year, resulting in unique flavours from a special mixing process	Available online from an Australian seller	Something I would have/give to my partner to show how much I care

Table S4: Questions included for segments stratification (responses between 1-2 = laggard, 3-4 = mainstream and 5-7 = lead user). Respondents had to respond to all questions with a number.

**Q1** - You consider yourself to be 'ahead of the curve' when it comes to newest ideas, brands, products for premium food products? - In this next section, we would like to ask you some questions about your purchasing and consumption behaviour of premium food products. There are no right or wrong answers, we are interested in your opinions. Please indicate the degree to which each statement is true or characteristic of you: (1=Not at all, 7=Extremely).
**Q2** - You are usually the first within your circle of friends to buy, try new products, brands for premium food products? - In this next section, we would like to ask you some questions about your purchasing and consumption behaviour of premium food products. There are no right or wrong answers, we are interested in your opinions. Please indicate the degree to which each statement is true or characteristic of you: (1=Not at all, 7=Extremely).
**Q3** - You try the newest brands because it insures you are getting the latest and most up-to-date options for premium food products? - In this next section, we would like to ask you some questions about your purchasing and consumption behaviour of premium food products. There are no right or wrong answers, we are interested in your opinions. Please indicate the degree to which each statement is true or characteristic of you: (1=Not at all, 7=Extremely).
**Q4** - You feel 'left out' if you're not quick to own newest items/services for premium food products? - In this next section, we would like to ask you some questions about your purchasing and consumption behaviour of premium food products. There are no right or wrong answers, we are interested in your opinions. Please indicate the degree to which each statement is true or characteristic of you: (1=Not at all, 7=Extremely).
Answer options for each question:
1
2
3
4
5
6
7

Figure S1: Example per category of how the respondent saw the concepts in the survey.

Which concept best fits your idea of the most premium/high end **BEEF**?
Please select one only

Option 1	Option 2	Option 3	Option 4
Slow cooked beef with onion and gravy, just heat and eat	Cuts like heart, liver, kidney... full of nutrition	Classic Beef Roast cut and tied, ready to cook	Beef sirloin, tender and juicy every time
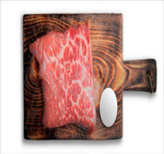	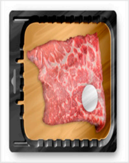	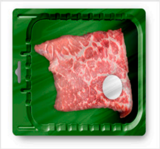	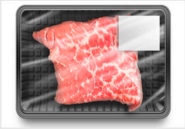
Traditional Australian Breeds like Brahaman or Angus	Unique breeds like older Longhorns that have a chance to develop more flavour, with a delicate beefy flavour and a slightly acid finish without having a very high fat content	Lean heart healthy beef, raised to have monosaturated fats to lower your blood pressure and cholesterol, but still have lots of flavour	Premium pasture fed beef from Blackmore's Wagyu, Cape Grim, or Minderoo
Highest quality premium meat, recommended by celebrities, and chefs as their favorite	Raised on a small family farm, grass feed using biodiverse pastures, hormone free, using sustainable farming practices	Aged using Traditional craftsmanship practices like dry aging for 35 days to tenderize and create a distinctive melt in your mouth flavour	Authenticated, traceable back to the farm
Available in the supermarket	Available at a small market / specialty store	Available online from a Chinese seller	Available online from an Australian seller
Something I would have/give to my partner to show how much I care	Something I would have for a special celebration	Something I would have for a special occasion like a festival or holiday	Something I would share when I am out with friends and family
○	○	○	○

Figure S1 cont.: Example per category of how the respondent saw the concepts in the survey.

Which concept best fits your idea of the most premium/high end **PORK**?

Please select one only

Option 1	Option 2	Option 3	Option 4
Fresh Australian pork loin chop	Tender and juicy Australian pork loin chop	Authentic Australian pork loin chop	Authentic, tender and juicy Australian pork loin chop
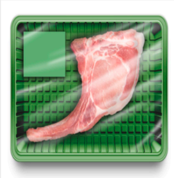	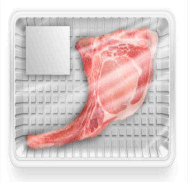	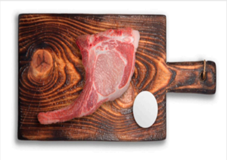	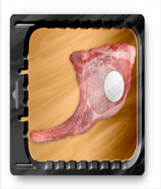
Healthy, natural Australian pork	Certified free range Australian pork	Locally sourced, tasty Australian pork	Traditional Australian pork farmed using sustainable practices
Australian pork packaged and prepared in China	Australian pork packaged and prepared in Australia	Fresh quality pork, 100% Australian farmed and butchered	Traditional European Berkshire breed. Farmed in Australia
Available at all stores where food and beverages are sold	Available at my local store	Available online with little information on country of origin	Available at my Supermarket
Something I would have by myself at home	Something I would share with friends and family	Something I would share when I am out with friends and family	Something I would have for a special celebration
○	○	○	○

Figure S1 cont.: Example per category of how the respondent saw the concepts in the survey.

Which concept best fits your idea of the most premium/high end **CHEESE**?

Please select one only

Option 1	Option 2	Option 3	Option 4
Premium quality	Certified organic	Flavorful cheese	Lightly flavored cheese
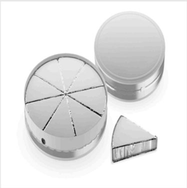	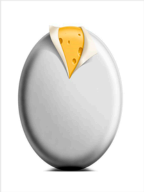	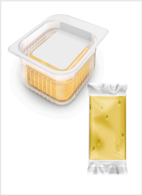	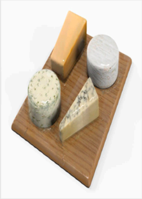
Made with fresh Australian A2 high protein milk	Made with Australian bush ingredients to enhance health and wellbeing	Made with fresh Chinese milk	Made with fresh Australian milk
Prepared in a modern clean facility	Authenticated, traceable back to the dairy farm	Hand crafted by a chef with a unique recipe	Handcrafted using traditional methods
Available at a small market / specialty store	Available online from a Chinese seller	Available in the supermarket	Available at my local store
Something I would have for a special celebration	Something I would have/give to my partner to show how much I care	Something I would have by myself at home	Something I would share with friends and family
○	○	○	○

Figure S1 cont.: Example per category of how the respondent saw the concepts in the survey.

Which concept best fits your idea of the most premium/high end **CHOCOLATE**?

Please select one only

**Option 1**	**Option 2**	**Option 3**	**Option 4**
Smooth plain slightly sweet milk chocolate	Full bodied dark chocolate	Premium quality	Certified organic
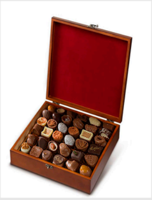	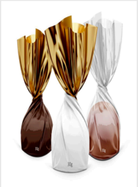	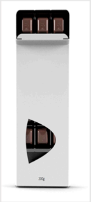	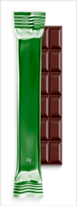
Cocoa from around the world is combined with Australian milk & sugar	Cocoa from around the world is combined with Chinese milk & sugar	Single origin globally sorced cocoa beans	Made from wild bush grown cocoa beans that provide the maximum health benefits from antioxidants
Handcrafted using traditional methods	Hand crafted by a chocolatier, with a unique recipe	Authenticated, traceable back to farms where the ingredients came from	Prepared in a modern clean facility
Available at all stores where food and beverages are sold	Available online from a Chinese seller	Available online from an Australian seller	Available at my local store
Something I would have for a special occasion like a festival or holiday	Something I would have/give to my partner to show how much I care	Something I would share when I am out with friends and family	Something I would have by myself at home
○	○	○	○

Table 5. Utility weight (regression coefficient) for beef, pork, cheese and chocolate concepts per attributes according to age, gender and country of origin (Australia or China),

	Country	Australia						China					
	**Gender**	**Male**			**Female**			**Male**			**Female**		
	**Age group**	**25-44**	**45-54**	**≥55**	**25-44**	**45-54**	**≥55**	**25-44**	**45-54**	**≥55**	**25-44**	**45-54**	**≥55**
	**Number of respondents**	**48**	**40**	**89**	**92**	**58**	**71**	**131**	**41**	**15**	**151**	**44**	**17**
**Attributes**	**Levels**												
**Product**	Beef mince with just the right amount of fat	-0.81	-0.42	-0.25	-0.43	-0.54	-0.63	-0.77	-0.64	-0.51	-0.65	-0.37	-0.63
	Beef stir fry, cut in just the right size	0.04	0.18	-0.20	-0.11	0.16	0.07	0.40	0.12	1.17	0.11	0.28	0.64
	Slow cooked beef with onion and gravy, just heat and eat	-1.11	-0.78	-0.37	-0.72	-0.92	-0.54	-0.15	-0.16	-0.02	0.00	-0.33	-0.37
	Cuts like heart, liver, kidney... full of nutrition	-2.49	-2.99	-2.69	-2.62	-2.65	-2.69	-0.55	0.02	-0.38	-0.11	-0.33	-0.23
	Classic Beef Roast cut and tied, ready to cook	1.60	1.42	1.36	1.61	1.42	1.35	0.24	0.03	-0.64	-0.01	0.13	-0.10
	Beef sirloin, tender and juicy every time	2.78	2.59	2.15	2.27	2.53	2.44	0.83	0.62	0.38	0.67	0.62	0.69
**Package**	Map tray sealed on top clear with loin cut	-0.22	-0.29	-0.20	-0.07	-0.16	-0.08	-0.77	-0.64	-0.51	-0.65	-0.37	-0.63
	Map tray sealed on top black with loin cut	-0.08	0.09	-0.01	0.06	-0.18	0.11	0.40	0.12	1.17	0.11	0.28	0.64
	Map tray seal with shiny black tray	-0.39	-0.50	-0.43	-0.50	-0.47	-0.44	-0.15	-0.16	-0.02	0.00	-0.33	-0.37
	Darfresh Vacuum sealed, green tray	0.04	0.16	0.10	0.01	0.09	-0.07	-0.55	0.02	-0.38	-0.11	-0.33	-0.23
	Matt black flat tray with tiny lip Darfresh vacuum, meat higher than lip with gold base	0.28	0.56	0.57	0.63	0.52	0.53	0.24	0.03	-0.64	-0.01	0.13	-0.10
	Butcher board square wood with meat on top	0.36	-0.03	-0.02	-0.13	0.21	-0.05	0.83	0.62	0.38	0.67	0.62	0.69
**Ingredient**	Fresh Australian beef	-0.39	0.18	0.30	-0.09	0.14	0.50	-0.27	-0.14	-0.23	-0.34	-0.17	-0.29
	Certified organic Australian beef	0.28	0.07	-0.09	0.35	0.15	0.28	0.30	0.17	0.48	0.28	0.28	0.71
	Traditional Australian Breeds like Brahaman or Angus	0.20	0.20	0.61	0.21	0.46	0.65	-0.29	-0.37	-0.67	-0.51	-0.59	-0.38
	Unique breeds like older Longhorns that have a chance to develop more flavour, with a delicate beefy flavour and a slightly acid finish without having a very high fat content	-0.33	-0.52	-0.63	-0.74	-0.85	-1.11	0.15	-0.04	-0.03	0.13	-0.03	-0.09

	Country	Australia						China					
	**Gender**	**Male**			**Female**			**Male**			**Female**		
	**Age group**	**25-44**	**45-54**	**≥55**	**25-44**	**45-54**	**≥55**	**25-44**	**45-54**	**≥55**	**25-44**	**45-54**	**≥55**
	**Number of respondents**	**48**	**40**	**89**	**92**	**58**	**71**	**131**	**41**	**15**	**151**	**44**	**17**
**Attributes**	**Levels**												
	Lean heart healthy beef, raised to have monosaturated fats to lower your blood pressure and cholesterol, but still have lots of flavour	-0.59	-0.46	-0.50	-0.28	-0.66	-0.55	0.34	0.57	0.71	0.58	0.71	0.72
	Premium pasture fed beef from Blackmore's Wagyu, Cape Grim, or Minderoo	0.84	0.52	0.31	0.54	0.77	0.23	-0.23	-0.18	-0.26	-0.15	-0.20	-0.67
**Provenance**	Australian meat packaged and prepared in China	-4.03	-4.58	-4.80	-4.70	-4.80	-5.39	-0.27	-0.12	0.03	-0.35	-0.16	-0.25
	Australian meat packaged and prepared in Australia	0.77	1.08	1.29	1.33	1.23	1.61	0.14	-0.08	-0.32	0.09	0.07	0.53
	Authenticated, traceable back to the farm	0.74	0.96	1.10	0.64	1.00	0.98	-0.01	0.41	-0.03	0.15	0.17	0.14
	Aged using Traditional craftsmanship practices like dry aging for 35 days to tenderize and create a distinctive melt in your mouth flavour	0.99	1.02	0.87	0.95	0.97	0.83	0.16	0.10	0.11	0.04	-0.01	-0.08
	Raised on a small family farm, grass feed using biodiverse pastures, hormone free, using sustainable farming practices	1.50	1.36	1.58	1.61	1.74	1.99	0.33	0.20	0.11	0.39	0.44	0.10
	Highest quality premium meat, recommended by celebrities, and chefs as their favorite	0.03	0.16	-0.03	0.17	-0.14	-0.02	-0.33	-0.51	0.10	-0.31	-0.51	-0.45
**Channel**	Available at all stores where food and beverages are sold	0.69	1.16	1.32	0.89	1.09	1.56	0.12	0.34	-0.27	0.20	0.10	0.53
	Available at my local store	1.45	1.43	1.75	1.69	1.50	1.86	0.10	0.03	0.96	-0.01	0.00	-0.14
	Available in the supermarket	1.34	1.33	1.57	1.37	1.62	1.83	0.59	0.40	1.01	0.67	0.71	0.34
	Available at a small market / specialty store	0.83	0.82	1.07	0.96	0.90	1.06	-0.71	-0.86	-0.93	-0.66	-0.65	-0.66
	Available online from a Chinese seller	-3.65	-4.07	-4.80	-4.42	-4.49	-5.28	-0.30	-0.38	-0.32	-0.49	-0.27	-0.28
	Available online from an Australian seller	-0.65	-0.67	-0.92	-0.49	-0.62	-1.03	0.22	0.47	-0.44	0.29	0.11	0.21
**Occasion**	Something I would have by myself at home	-0.41	0.06	0.00	-0.15	-0.25	-0.15	-0.08	-0.15	-0.30	-0.02	-0.08	0.01
	Something I would share with friends and family	0.73	0.61	0.61	0.58	0.77	1.01	0.42	0.25	0.13	0.27	0.31	0.19

	Country	Australia						China					
	Gender	Male			Female			Male			Female		
	Age group	25-44	45-54	≥55	25-44	45-54	≥55	25-44	45-54	≥55	25-44	45-54	≥55
	Number of respondents	48	40	89	92	58	71	131	41	15	151	44	17
**Attributes**	**Levels**												
	Something I would share when I am out with friends and family	0.11	0.17	0.01	0.17	0.06	0.22	-0.05	0.17	0.20	-0.06	0.21	0.14
	Something I would have for a special occasion like a festival or holiday	0.16	-0.05	0.03	-0.06	0.15	-0.32	-0.11	0.11	0.48	0.00	-0.28	-0.07
	Something I would have for a special celebration	-0.36	-0.53	-0.43	-0.29	-0.29	-0.49	-0.22	-0.25	0.07	-0.21	-0.25	-0.07
	Something I would have/give to my partner to show how much I care	-0.22	-0.26	-0.22	-0.26	-0.45	-0.26	0.05	-0.14	-0.56	0.01	0.10	-0.20

Table S5 cont.: Utility weight (regression coefficient) for **pork** concepts per attributes according to age, gender and country of origin (Australia or China).

	Country	Australia						China					
	**Gender**	**Male**			**Female**			**Male**			**Female**		
	**Age group**	**25-44**	**45-54**	**≥55**	**25-44**	**45-54**	**≥55**	**25-44**	**45-54**	**≥55**	**25-44**	**45-54**	**≥55**
	**Number of respondents**	**55**	**41**	**86**	**83**	**66**	**56**	**157**	**39**	**25**	**122**	**50**	**11**
**Attributes**	**Levels**												
**Product**	Pork loin chop	-0.46	-0.64	-0.70	-0.58	-0.53	-0.47	-0.46	-0.18	-0.69	-0.38	-0.63	-0.19
	Australian pork loin chop	-0.08	0.24	0.32	0.09	0.02	0.04	-0.25	0.04	-0.05	-0.27	-0.58	0.20
	Fresh Australian pork loin chop	0.10	0.23	0.43	0.35	0.07	0.29	-0.63	-0.63	-0.65	-0.57	-0.79	-0.67
	Tender and juicy Australian pork loin chop	-0.19	-0.03	-0.04	-0.17	-0.12	0.03	-0.23	-0.19	-0.08	-0.28	-0.15	-0.50
	Authentic Australian pork loin chop	0.17	0.10	-0.01	0.18	0.38	0.05	0.31	0.18	0.36	0.34	0.64	0.06
	Authentic, tender and juicy Australian pork loin chop	0.47	0.10	0.01	0.13	0.17	0.07	1.25	0.79	1.10	1.16	1.50	1.10
**Package**	Map tray sealed on top clear with loin cut	-0.17	-0.34	-0.04	-0.42	-0.21	-0.09	-0.06	-0.04	-0.40	-0.09	-0.07	0.13
	Map tray sealed on top black with loin cut	-0.16	-0.41	-0.40	-0.32	-0.02	-0.11	0.04	-0.11	0.74	-0.07	-0.29	-0.03
	Map tray seal with shiny black tray	0.45	0.51	0.25	0.51	0.59	0.39	-0.29	0.00	-0.08	-0.18	-0.10	-0.25
	Darfresh Vacuum sealed, green tray	0.70	0.57	0.37	0.63	0.51	0.15	0.21	0.04	0.26	0.19	0.07	-0.07
	Matt black flat tray with tiny lip Darfresh vacuum, meat higher than lip with gold base	-0.25	-0.25	-0.21	-0.39	-0.43	-0.40	0.51	0.40	0.20	0.42	0.49	0.15
	Butcher board square wood with meat on top	-0.56	-0.08	0.03	-0.01	-0.44	0.07	-0.41	-0.29	-0.72	-0.27	-0.10	0.06
**Ingredient**	Australian pork	-1.42	-1.39	-1.49	-1.13	-1.53	-1.43	-0.58	-0.69	-0.14	-0.65	-0.23	-0.82
	Fresh Australian pork, lean and heart smart	0.07	0.13	0.48	0.01	0.41	0.35	0.33	0.29	0.22	0.32	0.31	0.63
	Healthy, natural Australian pork	-0.75	-0.42	-0.27	-0.68	-0.57	-0.33	0.20	0.29	0.38	0.25	0.23	0.19
	Certified free range Australian pork	1.05	0.94	0.79	0.98	1.25	1.14	0.53	0.37	0.49	0.48	0.33	0.47
	Locally sourced, tasty Australian pork	0.32	0.30	0.27	0.43	0.19	0.03	-0.46	-0.43	-0.51	-0.43	-0.50	-0.25
	Traditional Australian pork farmed using sustainable practices	0.73	0.45	0.21	0.38	0.25	0.25	-0.01	0.16	-0.45	0.03	-0.14	-0.21
**Provenance**	Australian meat packaged and prepared in China	-5.39	-5.24	-5.46	-5.56	-5.63	-5.89	0.05	-0.17	-0.18	-0.21	0.45	0.19
	Australian meat packaged and prepared in Australia	1.05	1.19	1.02	0.87	0.82	1.37	0.15	-0.01	0.39	0.08	-0.02	-0.18
	Authenticated, traceable back to the farm	1.20	1.14	1.37	1.36	1.43	1.67	-0.28	0.18	-0.71	-0.05	-0.28	0.15

	Country	Australia						China					
	Gender	Male			Female			Male			Female		
	Age group	25-44	45-54	≥55	25-44	45-54	≥55	25-44	45-54	≥55	25-44	45-54	≥55
	Number of respondents	55	41	86	83	66	56	157	39	25	122	50	11
**Attributes**	**Levels**												
	Aged using Traditional craftsmanship practices like dry aging for 35 days to tenderize and create a distinctive melt in your mouth flavour	-0.25	-0.03	-0.23	-0.24	0.11	-0.40	-0.13	-0.22	0.02	-0.12	-0.28	-0.39
	Raised on a small family farm, grass feed using biodiverse pastures, hormone free, using sustainable farming practices	2.05	2.01	2.20	2.39	2.07	2.24	0.12	0.15	0.32	0.15	-0.01	-0.11
	Highest quality premium meat, recommended by celebrities, and chefs as their favorite	1.35	0.93	1.10	1.18	1.20	1.01	0.09	0.08	0.16	0.14	0.15	0.34
**Channel**	Available at all stores where food and beverages are sold	0.79	0.67	0.96	0.72	0.80	1.10	-0.26	-0.43	-0.53	-0.33	-0.15	-0.27
	Available at my local store	0.69	0.82	0.88	1.08	0.90	1.29	-0.01	0.24	0.10	0.17	0.27	0.28
	Available in the supermarket	-2.66	-2.69	-3.35	-3.21	-3.06	-3.53	-0.60	-0.82	-0.69	-0.73	-0.59	-0.82
	Available at a small market / specialty store	0.77	1.02	1.21	0.75	0.64	1.31	-0.16	-0.51	0.21	-0.51	-0.51	-0.83
	Available online from a Chinese seller	0.11	0.04	-0.12	0.15	0.22	-0.39	0.78	1.05	0.84	0.91	0.76	1.05
	Available online from an Australian seller	0.30	0.14	0.42	0.51	0.51	0.22	0.26	0.47	0.07	0.51	0.23	0.59
**Occasion**	Something I would have by myself at home	0.02	-0.13	0.13	-0.06	-0.02	0.08	-0.13	0.15	-0.67	0.21	0.11	-0.01
	Something I would share with friends and family	0.21	0.29	0.20	0.18	0.05	0.06	0.55	0.46	0.75	0.35	0.41	0.32
	Something I would share when I am out with friends and family	-0.07	-0.06	-0.10	-0.14	-0.16	-0.14	0.07	-0.06	0.33	-0.08	-0.15	-0.14
	Something I would have for a special occasion like a festival or holiday	0.14	0.02	0.00	0.10	0.20	0.04	-0.05	0.14	0.01	0.20	-0.31	0.07
	Something I would have for a special celebration	0.19	0.24	-0.08	0.23	0.28	0.04	-0.19	-0.21	-0.43	-0.22	-0.15	0.07
	Something I would have/give to my partner to show how much I care	-0.49	-0.36	-0.14	-0.31	-0.34	-0.07	-0.24	-0.49	0.00	-0.48	0.09	-0.32

Table S5 cont.: Utility weight (regression coefficient) for **cheese** concepts per attributes according to age, gender and country of origin (Australia or China).

	**Country**	**Australia**						**China**					
	**Gender**	**Male**			**Female**			**Male**			**Female**		
	**Age group**	**25-44**	**45-54**	**≥55**	**25-44**	**45-54**	**≥55**	**25-44**	**45-54**	**≥55**	**25-44**	**45-54**	**≥55**
	**Number of respondents**	**46**	**46**	**62**	**92**	**65**	**72**	**146**	**32**	**14**	**164**	**28**	**17**
**Attributes**	**Levels**												
**Product**	Premium quality	1.01	1.51	1.19	0.82	1.02	0.45	0.51	0.49	0.09	0.48	0.65	0.64
	Certified organic	0.16	-0.06	-0.15	0.13	0.28	0.35	0.41	0.23	-0.06	0.42	0.54	0.35
	Flavorful cheese	0.74	1.10	1.27	1.03	1.00	1.16	-0.24	0.01	-0.21	-0.33	-0.24	-0.25
	Lightly flavored cheese	-0.81	-0.86	-0.41	-0.71	-0.49	-0.31	-0.41	-0.31	-0.13	-0.32	-0.44	-0.65
	Cheese that is full of pieces of fruit or nuts	-0.81	-0.79	-0.90	-0.60	-0.74	-0.75	-0.15	-0.23	0.33	-0.19	-0.42	0.04
	A low lactose cheese that is delicious and easy on the tummy	-0.30	-0.90	-1.01	-0.68	-1.07	-0.90	-0.12	-0.19	-0.02	-0.07	-0.09	-0.11
**Package**	Slice cheese plastic individual wrap	-0.39	-0.94	-0.85	-0.91	-0.83	-0.74	0.06	0.18	0.10	0.05	-0.11	-0.22
	Cheese sticks	-0.96	-0.98	-1.14	-0.94	-0.98	-0.98	-0.29	-0.37	-0.10	-0.21	-0.17	-0.02
	Laughing cow triangle foil wrap in wheel	-0.31	-0.42	-0.38	-0.53	-0.23	-0.49	0.21	0.12	-0.11	0.10	0.14	-0.12
	Baby bell wax peel	0.23	0.48	0.54	0.47	0.33	0.61	0.36	0.32	0.08	0.51	0.63	0.21
	Tray platter with map packaging like catering for sharing	0.76	0.89	0.95	1.01	0.89	0.90	-0.12	0.02	0.13	-0.10	-0.09	0.09
	Cheese board with DarFresh vacuum pack	0.67	0.97	0.89	0.91	0.82	0.70	-0.21	-0.26	-0.09	-0.35	-0.40	0.07
**Ingredient**	Made with fresh Australian milk	1.31	1.54	2.12	1.57	1.99	2.15	-0.11	-0.55	-0.61	-0.18	0.11	-0.51
	Made with fresh Chinese milk	-4.26	-4.79	-5.16	-4.82	-5.45	-6.21	-1.19	-0.78	-0.90	-1.11	-0.80	-1.22
	Made with Australian bush ingredients to enhance health and wellbeing	1.19	1.04	0.74	0.99	1.14	1.14	0.78	0.72	0.56	0.78	0.39	0.60
	Made with fresh Australian A2 high protein milk	0.66	0.52	0.74	0.81	0.79	1.01	0.38	-0.07	-0.12	0.48	0.39	0.29
	Made with fresh milk, for the best tradition can offer	0.23	0.62	0.42	0.37	0.56	0.43	-0.38	-0.21	-0.16	-0.37	-0.58	-0.01
	Made with milk from a single cow free to roam on green pastures	0.87	1.06	1.15	1.09	0.97	1.48	0.51	0.89	1.23	0.41	0.50	0.86
**Provenance**	Prepared in a modern clean facility	-0.58	-0.69	-0.71	-0.93	-1.02	-0.86	-0.38	-0.12	-0.44	-0.40	-0.45	-0.19
	Authenticated, traceable back to the dairy farm	0.20	0.64	0.53	0.51	0.27	0.43	0.62	0.23	0.79	0.75	0.72	0.37

	Country	Australia						China					
	**Gender**	**Male**			**Female**			**Male**			**Female**		
	**Age group**	**25-44**	**45-54**	**≥55**	**25-44**	**45-54**	**≥55**	**25-44**	**45-54**	**≥55**	**25-44**	**45-54**	**≥55**
	**Number of respondents**	**46**	**46**	**62**	**92**	**65**	**72**	**146**	**32**	**14**	**164**	**28**	**17**
**Attributes**	**Levels**												
	Hand crafted by a chef with a unique recipe	-0.17	-0.09	0.16	0.03	-0.15	0.01	-0.62	-0.29	-0.05	-0.53	-0.91	-0.30
	Handcrafted using traditional methods	0.45	0.09	0.25	0.39	0.46	0.27	-0.27	-0.39	0.08	-0.12	-0.03	0.20
	Made with an uncompromising attitude to design and creation from years of expert knowledge	-0.53	-0.52	-0.56	-0.66	-0.53	-0.60	0.47	0.39	-0.20	0.07	0.25	0.11
	Limited small batches, prepared at the dairy, when all the ingredients are in peak season, quickly processed for a unique special flavor	0.63	0.57	0.33	0.66	0.95	0.75	0.18	0.19	-0.18	0.24	0.41	-0.18
**Channel**	Available at all stores where food and beverages are sold	1.05	1.32	1.39	1.22	1.57	1.36	0.11	-0.21	-0.46	0.15	0.32	0.30
	Available at my local store	0.72	0.81	0.91	0.77	0.97	0.97	-0.04	0.03	0.07	-0.25	-0.11	-0.20
	Available in the supermarket	0.74	0.61	1.30	1.06	0.84	1.32	0.10	0.47	0.43	0.12	0.30	0.36
	Available at a small market / specialty store	0.55	1.11	0.99	1.04	1.26	1.59	-0.29	-0.13	-0.23	-0.37	-0.42	-0.36
	Available online from a Chinese seller	-3.09	-3.80	-4.33	-4.08	-4.68	-5.40	-0.31	-0.34	-0.12	-0.31	-0.17	-0.57
	Available online from an Australian seller	0.02	-0.05	-0.26	0.00	0.04	0.17	0.42	0.18	0.31	0.65	0.07	0.47
**Occasion**	Something I would have by myself at home	-0.21	-0.56	-0.49	-0.40	-0.75	-0.62	-0.13	0.01	-0.12	-0.20	-0.43	-0.47
	Something I would share with friends and family	0.09	0.40	0.40	0.20	0.40	0.53	-0.31	-0.41	-0.56	-0.14	-0.10	-0.70
	Something I would share when I am out with friends and family	-0.08	0.05	0.01	0.02	0.09	0.30	0.54	0.70	0.64	0.52	0.51	0.51
	Something I would have for a special occasion like a festival or holiday	-0.33	0.07	0.04	-0.09	-0.10	-0.35	0.13	0.30	0.08	0.10	0.13	0.00
	Something I would have for a special celebration	0.64	0.27	0.44	0.51	0.48	0.42	-0.21	-0.47	-0.12	-0.32	-0.25	0.23
	Something I would have/give to my partner to show how much I care	-0.11	-0.22	-0.40	-0.23	-0.12	-0.29	-0.02	-0.13	0.08	0.04	0.14	0.43

Table S5 cont.: Utility weight (regression coefficient) for **chocolate** concepts per attributes according to age, gender and country of origin (Australia or China).

	Country	Australia						China					
	**Gender**	**Male**			**Female**			**Male**			**Female**		
	**Age group**	**25-44**	**45-54**	**≥55**	**25-44**	**45-54**	**≥55**	**25-44**	**45-54**	**≥55**	**25-44**	**45-54**	**≥55**
	**Number of respondents**	**136**	**44**	**66**	**155**	**49**	**78**	**144**	**40**	**15**	**143**	**40**	**14**
**Attributes**	**Levels**												
**Product**	Premium quality	0.46	0.37	0.37	0.33	0.42	0.37	0.85	0.75	0.69	0.78	0.71	1.18
	Certified organic	-0.03	-0.48	-0.11	-0.02	-0.26	-0.08	0.46	0.23	0.29	0.30	0.43	0.25
	Full bodied dark chocolate	0.00	0.34	0.20	0.13	0.21	0.41	-0.29	-0.26	0.07	-0.06	0.13	-0.35
	Smooth plain slightly sweet milk chocolate	-0.24	-0.23	-0.19	-0.26	-0.31	-0.30	0.05	0.33	-0.42	-0.28	-0.04	0.24
	Full of chewy, crunch nuts with the chocolate	0.17	0.10	-0.03	0.17	0.29	0.01	-0.55	-0.73	-0.10	-0.33	-0.77	-1.37
	Filled with a soft smooth filling	-0.37	-0.10	-0.25	-0.35	-0.36	-0.40	-0.53	-0.31	-0.53	-0.41	-0.45	0.06
**Package**	Silver wrap	-0.18	-0.46	-0.07	-0.35	-0.42	-0.17	-0.27	-0.23	-0.29	-0.26	-0.03	0.06
	Green wrap	-0.67	-1.22	-0.84	-0.95	-1.16	-0.78	-0.21	-0.36	-0.12	-0.25	-0.25	-0.46
	Pouch	-0.41	-0.90	-0.39	-0.32	-0.31	-0.23	-1.04	-0.96	-1.29	-1.12	-1.34	-1.02
	Blister pack	-0.54	-0.60	-0.47	-0.52	-0.59	-0.36	-0.05	0.05	0.13	-0.08	-0.16	-0.22
	Individual wrap	0.42	0.80	0.56	0.64	0.68	0.68	0.52	0.68	0.42	0.64	0.59	0.80
	Gift pack	1.38	2.38	1.22	1.50	1.81	0.86	1.05	0.81	1.14	1.08	1.19	0.84
**Ingredient**	Cocoa from around the world is combined with Australian milk & sugar	0.60	0.59	0.68	0.63	0.53	0.73	-0.19	-0.15	-0.19	-0.12	-0.35	-0.13
	Cocoa from around the world is combined with Chinese milk & sugar	-3.07	-2.80	-3.68	-3.49	-3.49	-4.26	-0.24	-0.46	-0.04	-0.41	0.24	-0.30
	Made from wild bush grown cocoa beans that provide the maximum health benefits from antioxidants	0.52	0.20	0.56	0.72	0.68	0.73	0.31	0.45	0.49	0.35	0.27	0.42
	Globally sourced cocoa blended with just a touch of sugar for a bitter chocolate, full of antioxidants	-0.10	0.43	-0.15	-0.18	0.08	-0.18	0.00	0.21	-0.06	0.12	-0.02	0.29
	Single origin globally sourced cocoa beans	0.04	-0.45	0.24	0.19	-0.02	0.24	-0.03	-0.25	-0.32	-0.27	-0.09	-0.39
	Australian cocoa beans blended with all Australian ingredients for a premium chocolate	2.01	2.03	2.35	2.14	2.22	2.74	0.14	0.20	0.13	0.33	-0.05	0.11

	Country	Australia						China					
	Gender	Male			Female			Male			Female		
	Age group	25-44	45-54	≥55	25-44	45-54	≥55	25-44	45-54	≥55	25-44	45-54	≥55
	Number of respondents	136	44	66	155	49	78	144	40	15	143	40	14
**Attributes**	**Levels**												
**Provenance**	Prepared in a modern clean facility	-0.88	-0.58	-0.68	-0.95	-0.81	-0.97	-0.37	-0.38	-0.83	-0.39	-0.30	0.41
	Authenticated, traceable back to farms where the ingredients came from	0.46	0.15	0.45	0.38	0.31	0.39	0.23	0.14	0.50	0.34	0.06	-0.10
	Hand crafted by a chocolatier, with a unique recipe	-0.04	0.08	0.00	0.10	0.21	0.14	0.28	0.09	0.39	0.17	0.34	-0.17
	Handcrafted using traditional methods	0.36	-0.01	0.29	0.29	0.11	0.46	-0.38	-0.24	-0.21	-0.59	-0.35	-0.22
	Made with an uncompromising attitude to design and creation from years of expert knowledge	-0.02	0.11	-0.05	-0.03	0.01	-0.09	-0.09	0.09	-0.06	0.02	0.04	-0.08
	Limited rare edition, cocoa beans are picked and roasted in 24 hours from the moment of peak timing once a year, resulting in unique flavours from a special mixing process	0.13	0.26	0.00	0.21	0.17	0.07	0.33	0.30	0.21	0.46	0.22	0.17
**Channel**	Available at all stores where food and beverages are sold	1.07	1.06	1.30	1.04	1.18	1.26	0.05	0.21	0.03	0.16	0.00	0.36
	Available at my local store	0.48	0.23	0.79	0.41	0.33	0.71	-0.18	-0.22	0.17	-0.19	-0.17	-0.42
	Available in the supermarket	0.92	1.34	1.34	1.02	1.48	1.50	0.57	0.33	0.69	0.57	0.61	0.35
	Available at a small market / specialty store	0.78	0.56	0.71	0.98	0.87	0.77	-0.58	-0.39	-0.74	-0.60	-0.54	-0.60
	Available online from a Chinese seller	-3.33	-3.07	-4.00	-3.52	-3.69	-4.10	-0.01	-0.10	-0.56	-0.23	-0.09	0.23
	Available online from an Australian seller	0.09	-0.11	-0.14	0.08	-0.17	-0.15	0.15	0.17	0.41	0.30	0.19	0.08
**Occasion**	Something I would have by myself at home	-0.61	-0.43	-0.71	-0.66	-0.61	-0.86	-0.45	-0.48	-0.25	-0.34	-0.35	-0.25
	Something I would share with friends and family	0.02	0.25	0.04	0.05	0.11	-0.01	0.05	-0.04	-0.16	0.24	0.34	0.06
	Something I would share when I am out with friends and family	-0.12	-0.25	-0.02	-0.14	0.09	-0.08	0.52	0.49	0.20	0.31	0.06	0.38
	Something I would have for a special occasion like a festival or holiday	0.06	0.03	0.25	0.17	0.11	0.37	-0.15	0.08	-0.02	-0.32	-0.21	-0.31
	Something I would have for a special celebration	0.77	0.58	0.67	0.66	0.59	0.67	0.04	0.00	0.05	0.00	0.07	-0.09

	Country	Australia						China					
	**Gender**	**Male**			**Female**			**Male**			**Female**		
	**Age group**	**25-44**	**45-54**	**≥55**	**25-44**	**45-54**	**≥55**	**25-44**	**45-54**	**≥55**	**25-44**	**45-54**	**≥55**
	**Number of respondents**	**136**	**44**	**66**	**155**	**49**	**78**	**144**	**40**	**15**	**143**	**40**	**14**
**Attributes**	**Levels**												
	Something I would have/give to my partner to show how much I care	-0.13	-0.20	-0.22	-0.08	-0.30	-0.09	-0.01	-0.07	0.19	0.11	0.08	0.21
